# Polyglutamine toxicity in yeast induces metabolic alterations and mitochondrial defects

**DOI:** 10.1186/s12864-015-1831-7

**Published:** 2015-09-03

**Authors:** Katharina Papsdorf, Christoph J. O. Kaiser, Adrian Drazic, Stefan W. Grötzinger, Carmen Haeßner, Wolfgang Eisenreich, Klaus Richter

**Affiliations:** Department Chemie, Lehrstuhl für Biotechnologie, Technische Universität München, Lichtenbergstraße 4, 85748 Garching, Germany; Department Chemie, Fachgebiet Elektronenmikroskopie, Technische Universität München, Lichtenbergstraße 4, 85748 Garching, Germany; Biological and Organometallic Laboratories, King Abdullah University of Science and Technology, Thuwal, 23955-6900 Kingdom of Saudi Arabia; Department Chemie, Fachgebiet Anorganische Chemie, Technische Universität München, Lichtenbergstraße 4, 85748 Garching, Germany; Department Chemie, Lehrstuhl für Biochemie, Technische Universität München, Lichtenbergstraße 4, 85748 Garching, Germany

**Keywords:** Polyglutamine, Iron-sulfur cluster, Mitochondria, Neurodegenerative disease

## Abstract

**Background:**

Protein aggregation and its pathological effects are the major cause of several neurodegenerative diseases. In Huntington’s disease an elongated stretch of polyglutamines within the protein Huntingtin leads to increased aggregation propensity. This induces cellular defects, culminating in neuronal loss, but the connection between aggregation and toxicity remains to be established.

**Results:**

To uncover cellular pathways relevant for intoxication we used genome-wide analyses in a yeast model system and identify fourteen genes that, if deleted, result in higher polyglutamine toxicity. Several of these genes, like *UGO1*, *ATP15* and *NFU1* encode mitochondrial proteins, implying that a challenged mitochondrial system may become dysfunctional during polyglutamine intoxication. We further employed microarrays to decipher the transcriptional response upon polyglutamine intoxication, which exposes an upregulation of genes involved in sulfur and iron metabolism and mitochondrial Fe-S cluster formation. Indeed, we find that *in vivo* iron concentrations are misbalanced and observe a reduction in the activity of the prominent Fe-S cluster containing protein aconitase. Like in other yeast strains with impaired mitochondria, non-fermentative growth is impossible after intoxication with the polyglutamine protein. NMR-based metabolic analyses reveal that mitochondrial metabolism is reduced, leading to accumulation of metabolic intermediates in polyglutamine-intoxicated cells.

**Conclusion:**

These data show that damages to the mitochondrial system occur in polyglutamine intoxicated yeast cells and suggest an intricate connection between polyglutamine-induced toxicity, mitochondrial functionality and iron homeostasis in this model system.

**Electronic supplementary material:**

The online version of this article (doi:10.1186/s12864-015-1831-7) contains supplementary material, which is available to authorized users.

## Background

During the last decades, protein misfolding and aggregation of certain proteins were found to play a major role in a variety of diseases, commonly called proteopathies [1–4]. For many of these diseases, age is a major risk factor. Some of them exhibit intracellular protein deposits, like in Parkinson’s disease, amyotrophic lateral sclerosis or Huntington’s disease, while others are diseases of the intra- and extracellular space, like systemic amyloidoses or Alzheimer’s disease [5]. In Huntington’s disease, Huntingtin (Htt), a protein with yet unattributed function, exhibits a tendency to aggregate within cells if mutated [6]. The age of disease onset and the aggregation propensity of Huntingtin are closely related to the length of a stretch of polyglutamine (polyQ) residues in its N-terminal domain [7]. The threshold to transform into a pathogenic protein was shown to be around 35–45 consecutive glutamine residues [8, 9]. Importantly, the phenomenon of polyQ aggregation is not limited to Huntington’s disease, but approximately a dozen other diseases are similarly associated with extended glutamine stretches in specific proteins [9–11].

The origin of toxicity of these aggregation-prone proteins is still under debate. One hypothesis states that the presumed toxic species ties all available chaperone activity to the aggregation process, thereby interfering with the protein quality control system. This would lead to a propagation of folding defects onto other cellular proteins [12–14]. In mammalian cells more than 200 proteins, including several chaperones, were identified in intracellular aggregates [15]. Experiments on model membranes also suggest that soluble oligomers of polyQ proteins are able to compromise the integrity of cellular membranes [16]. In addition, apoptosis and a purely mechanical constriction of neuronal axonal trafficking by aggregates has been suggested to be responsible for the loss of neurons [17–19]. The diversity of observations concerning the mechanism of pathogenesis highlights that potentially different cellular processes are affected in parallel.

The simplest and genetically most accessible eukaryotic model organism is *Saccharomyces cerevisiae*, in which several systems to study polyQ aggregation have been established [20–22]. Here diverse morphological effects have been described in response to polyQ-expression, including DNA-fragmentation, damage to respiratory chain complexes, apoptosis like effects, spindle formation defects, mislocalization of septin proteins and altered regulation of the prion state [*PSI*^+^] [20, 23–26]. In our model system, using Q_56_-YFP, the polyQ-induced cellular arrest phenotype (*pica*) is evident from small colony growth, enlarged cell size and incomplete septin assemblies during G_1_ phase prior to budding [20]. In contrast to other systems it is independent of the prion state of Rnq1 but dependent on the ploidy status of the yeast cell [20].

Comparing Q_0_-YFP and Q_56_-YFP expressing cells in this study we uncover further genetic interactors and analyze the transcriptional state of *pica* cells. In this process, we define genes, which help to reduce the toxicity of the polyQ protein and identify mitochondrial pathways, which likely are participating during establishment of toxicity.

## Results

### Q_56_-YFP toxicity is suppressed by a set of mitochondrial genes

**Fig. 1:**
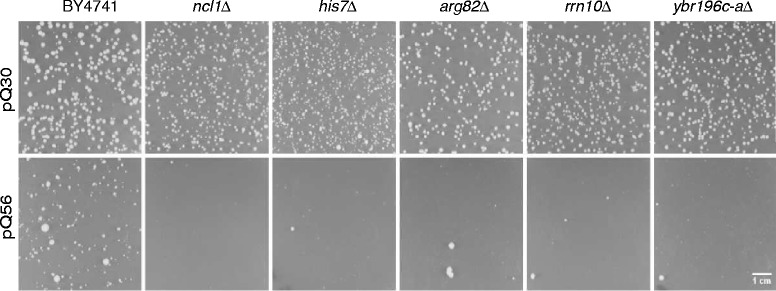
Selection of deletion strains unable to grow upon Q_56_-YFP expression. BY4741 and five deletion strains after transformation of either Q_30_-YFP (upper row) or Q_56_-YFP (lower row). The respective deletion is indicated on the top. Cells were grown on SD plates containing 2 % glucose. Growth was monitored after 4 days of incubation at 30 °C. Representative images are shown for selected strains (see Table 1 for full information). The scale bar represents 10 mm

In order to study polyQ induced toxicity we used a yeast model system, which consists of three different constructs fusing either zero, 30 or 56 glutamine residues to YFP [20]. In previous work the 56 amino acid stretch was found to be toxic, while the two other constructs were not harmful. Toxicity is evident from small colony growth after transformation with plasmids containing the constitutively expressed polyQ-encoding gene. To delineate the chain of events responsible for polyQ toxicity, we had performed a genome-wide screen of genomic deletion strains and identified yeast deletion mutants, which showed decreased toxicity in comparison to the wild type (WT) strain [20]. Using the same approach we now focused on deletion strains, which show increased toxicity. From 5160 strains, we retrieved fourteen knock-out strains, some of which had entirely lost the capability to form small colonies even after 15 days of incubation at 30 °C (Fig. 1, Table 1). The presence of these non-essential genes thus is required for the residual growth after pQ56 transformation.

**Table 1:** Suppressors of Q56-YFP toxicity

Gene knockout	Strain number	Gene function	Growth of Q_56_-YFP
*arg82*Δ	3531	Inositol polyphosphate multikinase (IPMK)	--
*atp15*Δ	1021	Epsilon subunit of mitochondrial ATP synthase	--
*cem1*Δ	198	Mitochondrial beta-keto-acyl synthase	--
*his7*Δ	3388	Imidazole glycerol phosphate synthase	---
*hom6*Δ	6933	Homoserine dehydrogenase	--
*ies2*Δ	1997	Essential for growth under anaerobic conditions	--
*kre28*Δ	4366	Subunit of a kinetochore-microtubule binding complex	--
*map1*Δ	5153	Methionine aminopeptidase	--
*ncl1*Δ	3050	S-adenosyl-L-methionine-dependent tRNA	---
*nfu1*Δ	4889	Protein involved in iron metabolism in mitochondria	--
*rpb9*Δ	4437	RNA polymerase II subunit	--
*rrn10*Δ	3051	Subunit of UAF (upstream activation factor) for RNA polymerase I	---
*ugo1*Δ	4304	Outer membrane component of the mitochondrial fusion machinery	--
*ybr196c-a*Δ	7457	Putative protein of unknown function	--

Genes, whose knock-out leads to an enhanced phenotype. The intensity of the phenotype is indicated on a semi-quantitative scale. --- : very strong toxicity, no residual growth, --: residual growth detectable, but stronger toxicity than in the WT background. Gene functions are annotated according to the yeast genome database

Most of the identified toxicity-suppressors participate in metabolic processes. Four of the 14 genes (*ATP15*, *CEM1*, *NFU1* and *UGO1*) are directly localized in mitochondria, one of the suppressors of toxicity (*IES1*) is required for anaerobic growth and at least 4 others are part of metabolic pathways shared between the cytosol and mitochondria (*HIS7*, *HOM6*, *MAP1*, *NCL1*). Thus, these deleted genes optimize essential systems, which are functional under normal growth conditions but become dysfunctional at conditions like polyQ intoxication. The increased proportion of mitochondrial genes indicates that in particular the mitochondrial system may be challenged in Q_56_-YFP producing yeast.

### Toxic and non-toxic polyQ stretches disturb the phosphate balance of the cells

To obtain more information on the condition of the *pica* yeast cells, we investigated the transcriptomic status of Q_56_-YFP intoxicated yeasts. We determined gene expression differences between intoxicated pQ56 and normally growing pQ0 transformed cells. We used four data sets to approach this question – Q_0__3d (pQ_0_ after 3 days), Q_0__2d, Q_56__3d and Q_56__4d – and obtained average relative expression changes for each gene (Additional file 1). We identified 76 genes, whose expression is reduced in pQ56 transformed cells to less than 33 % of the pQ0 transformed yeasts (Additional file 2).

**Fig. 2:**
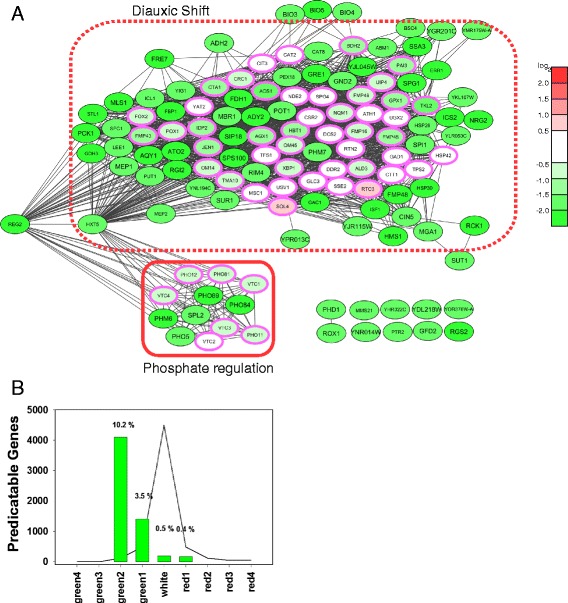
Reduced expression between Q_56_-YFP and Q_0_-YFP yeast cells. **a** Network of genes down-regulated in Q_56_-YFP cells compared to Q_0_-YFP cells. Genes are colored in accordance to their log differences (100 % green: log_2_ > 2, 75 % green: 2 > log_2_ > 1.5, 50 % green: 1.5 > log_2_ > 1, 25 % green: 1 > log_2_ > 0.5, white: 0.5 > log_2_ > −0.5, 25 % red: −0.5 > log_2_ > −1, 50 % red: −1 > log_2_ > −1.5, 75 % red: −1.5 > log_2_ > −2, 100 % green: −2 > log_2_). Hits with a p-value smaller than 0.05 were indicated by a greater font size (for details see Additional file 2). Pink frames highlight predicted co-regulated genes listed in Additional file 3. The smaller font size was additionally used for the predicted coregulators. For a detailed description of the data analysis see the methods section. Red boxes highlight clusters according to cellular pathways. **b** Analysis of predicted co-regulators within Fig. 2a. The line shows the number of genes within the respective category, while the vertical bar chart indicates the percentage of the genes predicted by the SPELL-correlations. If bars for all expression categories have the same height no significant enrichment in the predicted genes would be reported, which would indicate a non-significant clustering analysis

To define and visualize transcriptional clusters down-regulated in Q_56_-YFP expressing yeasts, we clustered our hits based on co-regulation patterns from co-expression databases [27]. In this way, most of the 76 genes down-regulated in the microarray experiments can be assembled into an interconnected network (Fig. 2a), as they apparently originate from two to three interconnected expression clusters. Beyond the initial hits we looked at further genes, which usually are part of these clusters: Using the SPELL database we automatically determined several co-regulated candidates with the highest connectivity and included them in the network of down-regulated genes (predicted genes are highlighted by a pink frame in Fig. 2a and listed in Additional file 3). This also helped to integrate further hits into the clusters of the network. Beyond that, these predicted candidates can be used to assess the predictive power of our network. Of the 50 candidates added to the network by the algorithm described in the methods section, 29 were in fact down-regulated to less than 60 % (log_2_ (Q_0_/Q_56_) > 0.5), while only 2 were up-regulated to the same extent (Fig. 2b), providing a sound statistical basis for the clusters formed in the network. It is evident from this network that transcripts related to phosphate metabolism and transport (*PHO5*, *PHO89*, *PHO84*, *SPL2*, *PHM6*, *VTC1*, *VTC2*, *VTC3*, *VTC4*) are down-regulated in Q_56_-YFP producing cells. This cluster had been observed before in a microarray study of yeasts expressing non-toxic polyQ proteins in liquid cultures [26]. The clear overlap to this study highlights that specific parts of the response to polyQ proteins are remarkably robust even if entirely different toxicity levels and growth conditions are examined. Beyond that, another large cluster of genes, which is usually up-regulated during diauxic shift, is lower expressed in Q_56_-YFP intoxicated cells when compared to Q_0_-YFP (Fig. 2a). This suggests that the normally growing pQ0 transformed cells in our study are undergoing diauxic shift whereas the pQ56 transformed cells do not, which might coincide with the smaller colonies formed or other metabolic abnormalities.

To see whether these expression differences coincide with toxicity, we determined differentially expressed clusters in response to the non-toxic Q_30_-YFP sample (Q_30__3d). Noteworthy, yeast cells synthesizing Q_30_-YFP, show no phenotype and are comparable to Q_0_-YFP expressing cells in terms of growth rate and colony size. Due to generally smaller differences in expression levels, we utilized a lower threshold – 0.6 log_2_ scales in both directions – to retrieve a reasonable number of hits for the analysis, while compromising on the p-value of the hits (Additional file 4). Correlations between the hits again were obtained from the co-regulation patterns in the SPELL database (Additional file 5A). Again further co-regulated candidates were derived from the SPELL database and incorporated into the network to evaluate the predictive power of the initial hit set (7 of these candidates were indeed down-regulated and 1 up-regulated of 16 predicted genes, Additional file 3 and Additional file 5B). Genes down-regulated in response to Q_30_-YFP form mostly one connected cluster. This cluster contains genes of phosphate metabolism and phosphate transport (*SPL2*, *PHM6*, *PHO5*, *PHO84*, *PHO89*, *VTC1*, *VTC2*, *VTC3*, *VTC4*). The consistent presence of this phosphate-cluster in all our experiments and in the previous study [26] is remarkable (Additional file 6A) and suggests that the presence of polyQ stretches results in alterations to phosphate regulation. Our data imply that this reaction may be a very sensitive marker in the reaction chain leading to the formation of the *pica* phenotype, but it is barely dependent on the toxicity level.

### Polyphosphate levels are elevated in Q_56_-YFP expressing yeasts

**Fig. 3:**
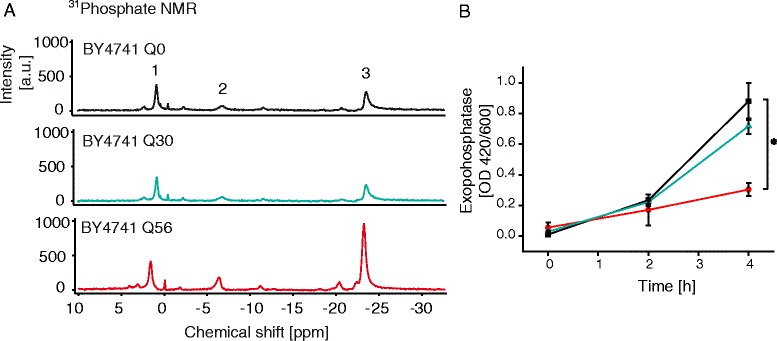
Effect of different polyQ stretches in yeast cells on phosphate metabolism. **a** ^31^P-NMR spectra of yeast cells expressing Q_0_-YFP (top), Q_30_-YFP (middle) or Q_56_-YFP (bottom). Yeasts were resuspended to an OD_595_ = 300 in phosphate free buffer. Chemical shift positions are listed in Additional file 7, 1: inorganic phosphate, 2: polyphosphate (end of chain), 3: polyphosphate (internal). **b** Exophosphatase activity was monitored upon transferring yeasts to phosphate free media. Q_0_-YFP is depicted in black, Q_30_-YFP in blue and Q_56_-YFP in red. Results were obtained from three independent experiments. Values were normalized for better comparability. Mean and SEM are depicted. Statistical significance is indicated with a star (α = 0.05)

We aimed at determining the metabolic impact of polyQ stretches to understand the cause for the differences in gene expression patterns. We thus analyzed whether dysregulation of the phosphate metabolism was evident in *pica* cells. To this end, we prepared suspensions of live Q_0_-YFP, Q_30_-YFP or Q_56_-YFP producing yeast cells and recorded ^31^P NMR spectra of these samples. Notably, distinct phosphate NMR signals could be detected in the cell suspensions (Fig. 3a; Additional file 7). Specifically, Peak 1 at 2 ppm was tentatively assigned to free phosphate, Peak 2 and 3 at −5.5 ppm and −24 ppm were tentatively assigned to polyphosphate [28–30], a storage form of phosphate, usually maintained by yeast cells in the vacuole and cytosol. The ^31^P NMR spectra of Q_0_-YFP and Q_30_-YFP producing yeast were virtually identical, whereas Q_56_-YFP expressing cells were different (Fig. 3a). Interestingly, the signal intensities for the inorganic phosphate were similar in all samples, implying that free phosphate levels are mostly comparable, whereas the signals originating from polyphosphate were increased by an approximate factor of 2 in pQ56 transformed yeast. This suggests that the phosphate balance is still maintained in Q_30_-YFP expressing yeasts despite the differences in induction of *PHO*-genes. In Q_56_-YFP expressing cells instead the phosphate homeostasis is misbalanced.

We aimed at determining the level of extracellular phosphatase activity by established assays in Q_56_-YFP, Q_30_-YFP and Q_0_-YFP yeast cells to see, whether the down-regulation of the extracellular phosphatases *PHO5*, *PHO11* and *PHO12* is observable on the protein level (Fig. 3b). Indeed Q_56_-YFP expressing yeast show reduced dephosphorylation activity compared to the two control strains, implying that the intoxicated yeast strains contain reduced amounts of phosphatases.

On this basis, it is tempting to speculate that the down-regulation of genes responsible for phosphate uptake and phosphate distribution in the cell could be related to the elevated polyphosphate levels in Q_56_-YFP synthesizing cells. Apparently in Q_30_-YFP the down-regulation of these genes enables the yeast cells to stabilize phosphate levels, while in Q_56_-YFP expressing yeasts the dysregulation manifests. Given that the transcriptional response already is observable in Q_30_-YFP producing yeasts, it is to be assumed that this part of the response is not related to toxicity.

### Expression changes in toxic Q_56_-YFP show a specific upregulated response

**Fig. 4:**
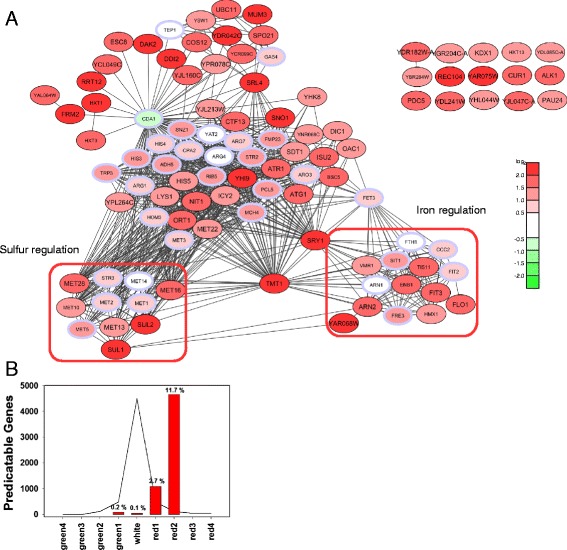
Enhanced expression between Q_56_-YFP and Q_0_-YFP yeast cells. **a** Network of genes up-regulated in Q_56_-YFP expressing cells compared to Q_0_-YFP expressing cells using the same color code as Fig. 2a. Hits with a p-value smaller than 0.05 were indicated by a greater font size (for details see Additional file 8). Blue frames highlight predicted co-regulated genes listed in Additional file 3. The smaller font size was additionally used for the predicted coregulators. Red boxes highlight clusters according to cellular pathways. **b** Analysis of predicted co-regulators within Fig. 4a. The line shows the number of genes within the respective category, while the vertical bar chart indicates the percentage of the genes predicted by the SPELL-correlations. If bars for all expression categories have the same height no significant enrichment in the predicted genes would be reported, which would indicate a non-significant clustering analysis

We then looked at the genes upregulated in the response to toxic Q_56_-YFP. Here we obtained 72 genes, whose expression is more than 2.5 fold higher in pQ56- compared to pQ0-transformed cells in different biological samples (Additional file 8), many of which show p-values with high significance (p < 0.05). For these genes we likewise determined a network of expression clusters utilizing the strategy outlined before (Fig. 4a). This network includes a cluster of sulfur-regulated genes, many of which are part of sulfur uptake and early steps of methionine metabolism (*SUL1*, *SUL2*, *STR3*, *MET1*, *MET16*, *MET8*, *HOM3*, *MET13*, *MET2*, *MET22*, *MET28*, *MET5*, *MET14*, *MET3*). Furthermore, a cluster of iron-regulated genes is up-regulated. This includes three of the four iron-transporters encoded in the genome, *ENB1*, *SIT1* and *ARN2* and genes related to iron-starvation (*FET3*, *FIT3*, *FIT2*, *TIS11* and *VMR1*). The sulfur and iron clusters are interconnected via several genes co-regulated with both clusters (Fig. 4a). We again determined co-regulated genes from the SPELL-database. Here, the connected and co-regulated candidate genes obtained from the SPELL database (highlighted with a blue frame) are indeed mostly up-regulated (26 of 33 predisctions are up-regulated, 1 down-regulated, Fig. 4b and Additional file 3). For many of them, the change in expression level was just below the 2.5 fold threshold for our initial hit list showing that many of the added genes are among the most strongly up-regulated genes within the data set (Additional file 3). Thus, from this microarray set we obtain upregulated expression networks with remarkable predictive power pointing to iron and methionine starvation in Q_56_-YFP producing yeast cells. Importantly, other stress responses, like the heat shock response, are not upregulated (see Additional file 9). Also nutrient supply appears sufficiently high despite the intoxication as evident from the down-regulation of clusters related to starvation (see Fig. 2a). Interestingly, the up-regulated genes do not overlap with earlier microarray studies on non-toxic polyQ [31]. They thus may indicate a potential correlation to toxicity.

We again looked at the Q_30_-YFP control cells to analyze, whether a similar response can be observed here. In these cells, though, only very few genes are up-regulated significantly (Additional file 10, Additional file 11A). Nevertheless we attempted to construct a connected network, but only few of the genes could be connected and only very few further genes could be uncovered from the SPELL database due to a general lack of connectivity. The predictive power of clusters in this Q_30_-YFP/Q_0_-YFP data set is much less pronounced (2 up-regulated and 1 down-regulated of 8 predicted genes, Additional file 3, Additional file 11B). Genes involved in iron and sulfur regulation are not retrieved, if Q_30_-YFP is used to stress the cell. This suggests that the alteration of iron and sulfur homeostasis is specific for *pica* cells exhibiting the growth phenotype (Additional file 6B).

**Fig. 5:**
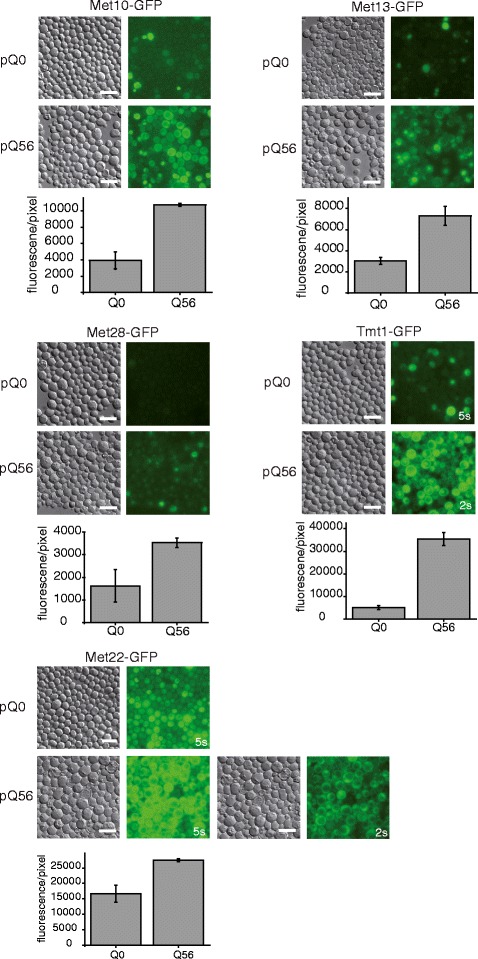
Up-regulation of Met10p, Met13p, Met22p, Met28p,and Tmt1p fused to GFP monitored on protein level. Exposure time is not changed between Q_56_-mCherry and Q_0_-mCherry cells if not stated otherwise. DIC and GFP pictures are shown. Quantification was performed with ImageJ. For a detailed description see methods section. GFP strains used in this work are listed in Additional file 16. Scale bar represents 10 μm

We tested the up-regulation of some genes from the iron- and sulfur-clusters by investigating genomic GFP-fusions of the respective proteins in the Q_56_-mCherry intoxicated and Q_0_-mCherry control background. Indeed for several of them, like Met10p, Met13p, Met22p, Met28p and Tmt1p we find a marked up-regulation observable by fluorescence microscopy of the GFP-fused proteins (Fig. 5), implying that the up-regulation is also evident at the level of protein concentrations within the cell.

### Iron-homeostasis is affected in Q_56_-YFP producing yeasts

**Fig. 6:**
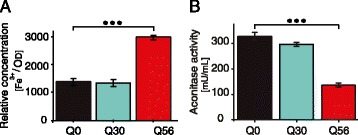
Iron concentration and aconitase activity in Q_56_-YFP and Q_0_-YFP yeasts. **a** Relative concentration of intracellular free iron in yeast cells expressing Q_0_-YFP, Q_30_-YFP or Q_56_-YFP determined by EPR spectroscopy. Yeasts were treated with DETAPAC and DFO to complex iron and resuspended to an OD_595_ = 300 in HEPES buffer. Results are expressed as mean ± SEM (n ≥ 3). **b** Aconitase activity of yeast cells expressing Q_0_-YFP, Q_30_-YFP or Q_56_-YFP was determined colorimetrically. Results are expressed as mean ± SEM (n ≥ 3). Statistical significance is indicated by three stars (α = 0.005)

Having observed a strong up-regulation of iron and sulfur associated expression clusters in *pica* yeast we tested, whether a lack of iron ions causes this response. We determined the total intracellular free iron concentration in intact cells by electron paramagnetic resonance, (EPR) (Additional file 12). Membrane-permeable desferroxiamine (DFO) was used to complex free Fe^3+^ and oxidize intracellular Fe^2+^ [32, 33]. Surprisingly, Q_56_-YFP cells contain two-fold higher levels of intracellular iron compared to the control cells. Cells producing Q_30_-YFP instead show no alteration of the free iron level (Fig. 6a). The strong increase of intracellular iron levels in pQ56 transformed *pica* cells reflects a clearly disturbed intracellular iron balance. The higher levels of free iron though seem to contradict the observed up-regulation of iron-transporters. However, iron-regulation and its metabolism are both very complex and closely tied to the synthesis of Fe-S cluster proteins. In fact, up-regulation of iron-regulated genes as observed in Q_56_-YFP yeast is usually induced via the transcription factor Aft1, if Fe-S clusters are not produced to sufficient extent in mitochondria [34, 35].

To evaluate, whether *pica* yeasts show deficiencies in the generation of Fe-S cluster proteins in general, we tested the activity level of the prominent Fe-S cluster protein aconitase. While Q_0_-YFP and Q_30_-YFP producing yeast cells show very similar aconitase activities, we observed a strong reduction in aconitase activity in Q_56_-YFP yeasts (Fig. 6b). Hence, the higher level of free iron in the cell is apparently not sufficient to ensure the production of the Fe-S cluster containing enzyme aconitase. The reduced production of Fe-S cluster proteins in the mitochondria of *pica* yeast might thus cause the up-regulation of the iron-regulatory expression cluster observed.

### Q_56_-YFP reduces mitochondrial carbon source utilization

**Fig. 7:**
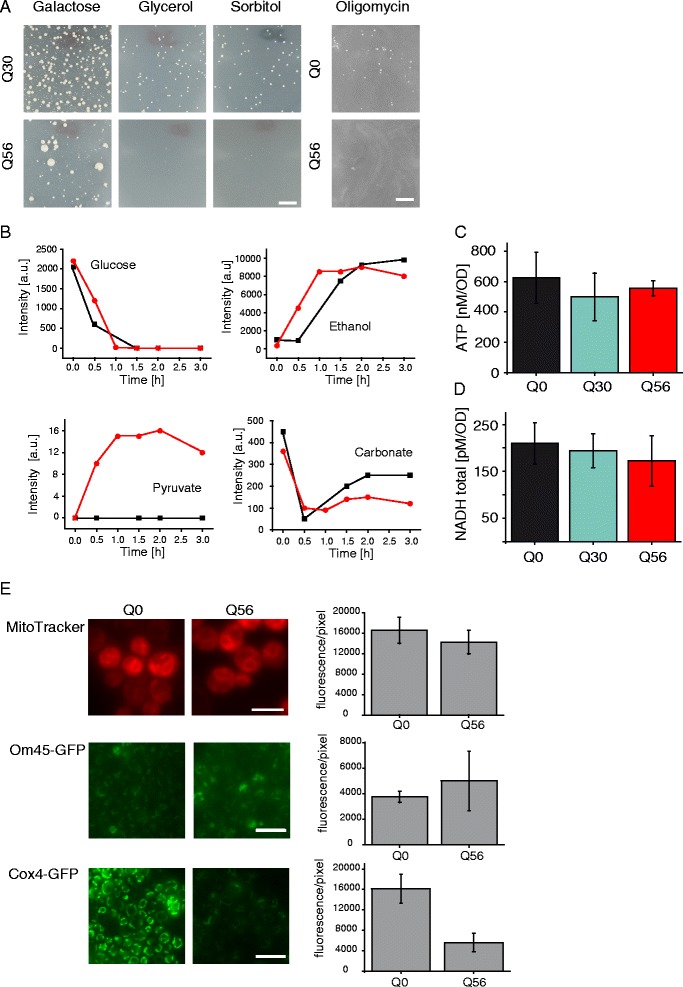
Alteration in carbon metabolism upon polyQ intoxication. **a** Growth of transformed yeasts was monitored after 8 days of incubation. The *pica* phenotype of Q_56_-YFP is enhanced when BY4741 cells are grown on SD media containing 2 % sorbitol or 2 % glycerol. The *pica* phenotype is unchanged if cells are grown on 2 % galactose as a carbon source compared to glucose. Upon respiratory chain inhibition by adding oligomycin no growth is observable after pQ56 transformation. The scale bar represents 10 mm. **b** Yeast were resuspended to an OD_595_ = 150 and [U-^13^C_6_]-glucose was added at time point 0. Spectra were recorded for 3 h by NMR. The kinetics of glucose consumption are based on the peak at 75.8 ppm and ethanol production based on the peak at 16.7 ppm in pQ0- (black square) and pQ56- (red circle) transformed yeasts. The kinetics of pyruvate accumulation are based on the peaks at 169.9 ppm and carbonate production quantified based on the peak at 160.3 ppm in pQ0- (black square) and pQ56- (red circle) transformed yeasts. The chemical shift positions are assigned in Additional file 13 and Additional file 14F and G. **c** ATP-level of Q_0_-YFP, Q_30_-YFP and Q_56_-YFP yeasts was determined using a luciferase coupled assay. Means and SEM are depicted. Six biological replicates were analyzed. The detected differences are not significant. **d** NADH-level of Q_0_-YFP, Q_30_-YFP and Q_56_-YFP yeasts. The detected differences are not significant. **e**) MitoTracker staining in Q_56_-YFP and Q_0_-YFP expressing cells. Expression time is not changed between samples. Scale bar represents 5 μm for MitoTracker. Expression pattern of Cox4p and Om45p fused to GFP. Exposure time is not changed between Q_56_-mCherry and Q_0_-mCherry producing cells. Scale bar represents 10 μm for Cox4-GFP and Om45-GFP. Quantification was carried out as described in the methods section. Mean fluorescence per pixel is plotted with SD

We had found several indications that the mitochondrial system in *pica* cells is compromised and related to toxicity. To get direct information on the ability to perform non-fermentative metabolism, transformed yeast samples were placed on SD media containing 2 % sorbitol or 3 % glycerol instead of 2 % glucose [36, 37]. Both alternative carbon sources make yeasts more dependent on respiration compared to glucose and require functional mitochondria. As a control, we used galactose, which can be metabolized in a similar manner as glucose. When transformants of control plasmids pQ0 or pQ30 were plated on 3 % glycerol medium, growth was generally slower compared to glucose plates, but the colony patterns after four additional days of incubation resembled those of yeasts grown on glucose (Fig. 7a). Q_56_-YFP cells instead behaved differently. While few of the large colonies grew, which are resistant to the *pica* phenotype due to polyploidization or shortened polyQ stretches [20], the vast amount of small colonies is absent. Also, on 2 % sorbitol the growth suppression by Q_56_-YFP expression was stronger leading to the absence of small colonies (Fig. 7a). Usage of galactose as carbon source instead did not lead to absence of *pica* colonies, implying that the residual growth of these cells can be maintained, as long as carbon sources are used that allow sufficient energy production under fermentative conditions. To further study the importance of respiratory activity for growth of *pica* yeast we inhibited the respiratory chain by cultivating them on oligomycin. Indeed, we observed a loss in growth of pQ56 transformed yeasts whereas the pQ0 cells were still viable at the same oligomycin concentration (Fig. 7a).

To determine whether also differences in mitochondrial activity can be observed on the metabolic level, we tested the capability of *pica* yeasts to metabolize glucose. To this end we added [U-^13^C_6_] glucose to yeast suspensions of Q_0_-YFP or Q_56_-YFP cells and analyzed the samples by ^13^C-NMR spectroscopy at different time points after addition of the substrate (Fig. 7b). Within two hours control yeast produce metabolites of different kinds including ethanol, whereas the signal sets for glucose are diminished (Additional file 13, Additional file 14). The same signal patterns are observed with suspensions of pQ56 transformed yeast cells demonstrating that the conversion of glucose in the fermentative pathway in general is not affected by the presence of extended polyQ stretches (Fig. 7b). Looking into the weaker signals it becomes obvious that throughout the time course of the measurement small peak sets, which based on the chemical shifts and the J-couplings can be attributed to pyruvate, are observable only in pQ56 transformed suspensions (Fig. 7b and Additional file 14F and G). Further, a slight reduction in the carbonate peak at 160.3 ppm is evident in pQ56 transformed yeasts (Fig. 7b). This difference confirms an impaired respiratory activity of mitochondria as apparently the respiratory conversion of pyruvate to CO_2_ is reduced in the *pica* cells.

### Q_56_-YFP intoxication results in decreased Cox4p levels

We aimed at determining, whether the altered metabolic pathways induce the growth arrest by energy shortage, or whether other mitochondrial functions, like generation of Fe-S cluster proteins, are more relevant for the *pica* phenotype. To this end, we investigated ATP-levels of Q_56_-YFP cells and normal growing Q_0_-YFP cells. Despite the reduced metabolic activity, ATP-levels themselves seem not to be altered in *pica* yeasts compared to control yeasts (Fig. 7c). We further investigated NADH levels, as these also could be misbalanced due to their relationship to mitochondrial metabolism. The detected differences in NADH level between *pica* and control yeasts are minor and not significant (Fig. 7d). Even though these values relate to full cell lysate and not isolated mitochondria, they imply that energy metabolism is still working in these yeasts. We finally aimed at testing by fluorescence microscopy whether proteins within the mitochondria are affected by the polyglutamine intoxication, which could also explain the deficiency in aconitase and the up-regulation of iron-regulated genes. Based on staining with the dye MitoTracker the mitochondrial system seems to be intact and the tubular network is formed (Fig. 7e). Two mitochondrial proteins were analyzed then by fluorescence microscopy, Om45 and Cox4. Both are slightly lower expressed in Q_56_-YFP yeasts based on our microarray analysis (−0.96 log_2_ and −0.75 log_2_ respectively). The mitochondrial membrane protein Om45 does not detectably change its cellular localization or its protein levels (Fig. 7e). Looking at Cox4p, a component of the respiratory chain, instead, the protein levels are strongly diminished in Q_56_-YFP expressing yeasts, implying that at the level of distinct proteins alterations to the mitochondrial system become observable (Fig. 7e). These data suggest that, while mitochondria generally appear functional in respect to energy metabolism, alterations within the mitochondria of *pica* cells become observable in connection to toxicity.

## Discussion

Elongated polyQ stretches have been reported as genetic cause for several neurodegenerative diseases, including Huntington’s disease [20, 22, 38, 39]. The reasons for this toxicity and the cellular pathways relevant for this process are under debate and many alterations have been reported, including DNA-fragmentation, apoptosis, spindle disorganization and involvement of the chaperone system or diverse prion proteins [20, 23, 24, 26]. Here we used a toxic polyQ system composed of zero, 30 or 56 glutamine residues to reveal affected cellular pathways based on an un-biased approach starting from a genome-wide screen of the EUROSCARF deletion strain library and a genome-wide assessment of expression changes upon intoxication.

Fourteen genomic deletion strains were uncovered that act synergistically with the polyQ induced phenotype. The knock-out of these genes prevents the residual growth, which in the wild type background is evident from many small colonies on agar plates. Interestingly, the majority of these genes are associated with mitochondrial functions, including the mitochondrial fusion mediator Ugo1p, the subunit of the F_1_F_0_-ATPase Atp15p and the protein Nfu1p, which is involved in Fe-S cluster synthesis. Several of these genes, including *UGO1* and *ATP15*, have before been associated with the *petite*-phenotype, a small-colony formation phenotype, which can be intensified by cultivation on non-fermentative growth media. Given the similar appearance of polyQ-intoxicated *pica* yeasts, a related cause for these phenotypes could be speculated on, in particular as *pica* responds to the growth on non-fermentative carbon sources likewise with a general inability to form any colonies [40]. Our screening approach had excluded strains with significant growth defects due to their genomic deletion, in particular when residual growth of small colonies could be observed after pQ56-transformation. We thus omitted some strains, which show the *petite*-phenotype, even though they were retained in the first round of screening. Two of these strains have deletions in MGM1 and FZO1. Given that UGO1, MGM1 and FZO1 are functionally related in mitochondrial fusion we reanalyzed their behavior in response to pQ56-transformation (Additional file 15). Indeed the differences in colony pattern between Q_0_-YFP expression and Q_56_-YFP expression suggest growth defects that are stronger than the additive effects of mutation and Q_56_-YFP toxicity.

The transcriptional profiling reported here additionally uncovers pathways with mitochondrial association and suggests the iron- and sulfur-metabolism as a potential target. Both pathways converge in the mitochondria during Fe-S cluster synthesis [34, 41]. We observe here the up-regulation of a transcriptional cluster, which is usually under the control of the transcription factor Aft1. This cluster includes the iron importers in the plasma membrane and several other proteins, which regulate the distribution and metabolism of iron ions in the cell. Generally this transcriptional cluster is induced by the lack of or shortage of Fe-S cluster proteins via the Fe-S containing sensor complex Grx [42, 43]. This reduction becomes obvious in our system by the reduced activity of aconitase, a very prominent Fe-S cluster protein in the cytosol. This shortage is not due to down-regulation of aconitase as evident from our genome-wide expression data, which show only slightly up-regulated ACO1 and slightly down-regulated ACO2 expression, but is apparently due to reduced levels of active protein (ACO1: −0.35 log_2_; ACO2: 0.52 log_2_ ). The increased levels of iron in the cell do not necessarily have to imply that Fe^2+^ is sufficiently present in the mitochondria for generation of Fe-S clusters. Fe^3+^ could also be present in the cytosol or even in complex with polyphosphate in the vacuole, all of which could lead to a shortage of mitochondrial iron in the presence of excessive Fe^3+^ [44]. Likewise the accumulation of polyphosphate could be caused by the accumulation of iron in the cells given that these two are found in complex with each other on multiple occasions. It is interesting to note that Cox4p, a subunit of the cytochrome C oxidase complex (Complex IV) in the mitochondria and highly sensitive to polyQ intoxication, is part of a protein complex, which complexes iron ions [45]. Thus proteome-wide data may indeed be necessary to establish, whether in particular iron-containing proteins are affected by the presence of polyQ containing aggregates. It is very interesting to further see, that the highly responsive heat shock and stress response network of chaperones does not react in polyQ intoxicated cells. This could indeed indicate that this part of the proteostasis network is not directly involved or at least that shortage of chaperones is not causative to the toxicity experienced in our yeast model system.

Several genome-wide studies had been performed in the past on similar aggregation systems in higher eukaryotes [46–50]. These uncovered a broad range of genetic and physical interactors. As such an RNAi screens in *C. elegans* uncovered metabolic influences, but in connection with the ascorbate and aldarate system [46]. Additionally the protein homeostasis system was shown to suppress polyglutamine aggregation [51]. Transcriptional changes in huntingtin mice and Huntington's patients point to genes in signal transduction [47], likewise do studies in mammalian cell culture based on RNA interference [48, 49]. Also studies in *Drosophila melanogaster*, employing huntingtin and ataxin as model systems find genes in signal transduction and cellular proteostasis [50].

Beyond that, the damage to the mitochondrial system has been observed in other aggregation model systems repeatedly [52, 53]. Also in subsets of patients suffering from neurodegenerative diseases the involvement of mitochondrial damage or the involvement of iron-metabolism has been encountered [54, 55]. In several cases the reduction in aconitase and the reduction in respiratory chain complexes have been observed [25, 56]. Another striking example linking the iron-metabolism to neurodegenerative disease is the protein frataxin, which itself is the iron-importer into mitochondria. This protein, if its polyQ stretch is expanded, tends to aggregate and result in neurodegenerative ataxias [57]. While it is speculative to assume a common mechanism in generation of neurodegenerative defects in polyQ diseases, it emerges that alterations and damages to the mitochondrial system are highly relevant for the toxicity observable upon expression of polyQ proteins also in yeast.

## Conclusion

Protein aggregation of elongated polyglutamine stretches induces cellular defects and death. Analyzing the transcriptional response in yeast we found two clusters connected to the iron and sulfur metabolism to be up-regulated in presence of extended toxic polyglutamine stretches. We report an accumulation of iron *in vivo* and a reduction in the activity of the prominent Fe-S cluster containing enzyme aconitase. In a genome wide approach, genes related to mitochondrial proteins were found to be crucial for cellular survival. Our data suggest an intricate connection between polyglutamine-induced toxicity, mitochondrial functionality and iron homeostasis in this model system.

## Methods

### Yeast cultivation and storage

*S. cerevisiae* strains were generally cultivated at 30 °C. Media were chosen according to the selection requirements. WT as well as knockout strains were grown on YPD-plates or in YPD liquid cultures [58]. Strains transformed with plasmids pQ0, pQ30 or pQ56 were grown on appropriate SD media plates. All carbon sources were added at a concentration of 2 % (w/v) except for glycerol, which was added at 3 % (v/v). If not explicitly stated otherwise, the carbon source was glucose. The respiratory chain inhibition was performed by adding 8 mg/ml of an oligomycin isomers mixture (Merck Chemicals GmbH, Schwalbach, Germany) to the SD media plates.

The haploid Saccharomyces Genome Deletion Project library (EUROSCARF, Frankfurt, Germany) in the BY4741 background (*MAT***a**; *his3Δ1*; *leu2*Δ*0*; *met15*Δ*0*; *ura3*Δ*0*) was used for screening. Screening conditions were as described previously [20]. In the first round of screening, we retained all strains, growing weakly after pQ56-transformation. In subsequent rounds, we discarded strains, which show a strong growth defect by themselves due to the genomic deletion in cases, where residual growth of small *pica* colonies was observed, as under these conditions it is difficult to clearly distinguish between synergistic and additive effects. For the analysis of cellular structures affected by the presence of polyQ proteins, the respective strains of the Yeast GFP fusion collection in the background of EY0986 (*MAT***a**; *his3Δ1*; *leu2Δ0*; *met15Δ0*; *ura3Δ0*) were deployed [59]. All GFP-strains used in this manuscript are listed in Additional file 16.

### Yeast transformation

Yeast transformations were performed using an adapted version of the simplified lithium acetate transformation method [60]. The same transformation protocol was used for single transformations as well as for transformations in the 96-well format. 200 μl of a 2 day culture were spun down gently and resuspended in PLATE-solution (40 % PEG4000, 100 mM LiOAc, 10 mM Tris/HCl pH 7.5, 1 mM EDTA, 46 mM DTT). 5.1 μg/ml salmon carrier DNA were added together with 100 ng of plasmid DNA. The mixture was incubated at room temperature for 16 h and subsequently a heat shock was performed for 1 h at 42 °C. The transformation mixture was plated onto appropriate minimal medium and incubated at 30 °C. For 96-well transformations PLATE, salmon carrier DNA, DTT and plasmid were part of a premix to resuspend the yeast cell pellet.

### Growth for GeneChip experiments

To analyze gene expression changes in response to polyQ proteins of different lengths, yeast samples were washed directly off the transformation plates. Plates bearing pQ56 transformants contain mostly small colonies. The few large colonies found on these plates were removed before harvesting the cells. As colony growth rates also correlate with carbon source consumption, yeasts were analyzed after three or four days on plates. Several plates had to be harvested for Q_56_-YFP after 3 days (Q56_3d) or Q_56_ after 4 days (Q_56__4d) due to small colony size to obtain the same amount of biomass, while one plate was sufficient for the samples Q_0__2d, Q_0__3d and Q_30__3d. Cells were pelleted for 10 min at 2500 x g and shock frozen in liquid N_2_. The isolation and enrichment of mRNA and further preparation including microarray analysis on Affymetrix GeneChip Yeast Genome 2.0 [61] were carried out by the Kompetenzzentrum für Fluoreszente Bioanalytik (Regensburg, Germany). The GeneChip Yeast Genome 2.0 contains 5744 probe sets for genes of *S. cerevisiae* and 5021 probe stets for genes of *Schizosaccharomyces pombe*.

### Hit selection from microarrays

The microarray data originated from three experimental sets. Q_0__3d, Q_30__3d and Q_56__3d were processed together, likewise Q_0__2d and Q_56__4d. A further set of Q_0__3d and Q_30__3d was generated as replicate. The data provided by the Kompetenzzentrum für Fluoreszente Bioanalytik had already been normalized within one assay set, using the multi-chip analysis (RMA) algorithm [62, 63] and MAS5 [64]. Generally the p-values for the individual hits were < 0.05, as long as the signal intensity of the MAS5 data was >15. All raw data sets are provided together with this manuscript and will be available to the public from www.richterlab.de.

To obtain relative expression differences between two samples, we used the MAS5 value for each gene, calculated the ratio (e.g. Q_0__3d/Q_56__3d) and sorted the full data set of 5900 probes regarding this value. The least-affected gene should then be at position 2950 with a quotient of 1. Thus the ratio column was divided by the median value obtained from position 2950. This approach was also used in the other data sets for normalization. All ratios were then converted to logarithmic values.

For the comparison of Q_0_-YFP and Q_56_-YFP samples, all possible combinations of the data sets were calculated and included. From the relative logarithmic expression differences we determined the average expression change and the standard deviation. The full set was then sorted according to this average relative expression change. Genes were included in the final hit list, if they showed expression differences of at least threefold (Q_56_ down versus Q_0_), 2.5 fold (Q_56_ up versus Q_0_) or 1.5 fold (Q_30_-YFP versus Q_0_-YFP, both directions). Expression changes below these values were not included in the hit list.

To define which signal intensities correspond to noise levels we used the *S. pombe* spots on the array as these probes will not detect specific genes and consequently define noise. This correlates well with the fluctuations in data with very low MAS5 values. Genes below this noise level (MAS5 values of 15) were generally not considered to be hits. During all these calculations no further genes were manually removed or edited.

### Cluster analysis

All hits above noise level for either up- or down-regulation were included in the cluster analysis. To this end, we obtained the 20 highest ranking co-regulated genes for each of our hits from the SPELL database, which provides this information based on the analysis of more than 10,000 microarray data sets [27]. Being listed together within these 21 genes (original gene plus 20 coregulators) was considered an incidence of co-regulation. A pairwise co-regulation matrix was built including the information from all the hits. Highly correlated genes were observed with up to 25 connections between each other. This matrix was used to draw a map with the open-source software CytoScape [65, 53]. The layout function “Edge-weighted Spring Embedded” was used for initial visualization of the clusters and the final maps were obtained by moving the nodes to prevent graphical overlap for better visualization.

In the likely case, that among the 20 co-regulated genes obtained from the SPELL database for a single hit genes showed up that were not in the hit list, a non-hit was included in the co-regulation matrix and if this non-hit was found co-regulated more often these co-regulated non-hits could obtain high levels of connectivity within our network. To test the significance of our network we picked those non-hit genes from our co-regulation matrix, which are best connected within the network and considered them to be part of the clusters in our network. If they are part of the clusters, they should also be regulated together with the cluster in our microarray data set, but possibly these genes were differentially expressed to an extent just below our inclusion threshold. To test, whether this is the case we obtained the experimental expression values for these “predicted further co-regulated genes” from our microarray data and tested, whether they are regulated in the appropriate direction in our experimental data set. If significantly more of these predicted genes are regulated together with the hit-based network than opposite to it, it would validate the predictive quality of the network and thus the validity of the clusters determined. For implementing this algorithm we used the integrated development environment Dev C++ 4.9.9.2 (Bloodshed Software, www.bloodshed.net). For all figures in this manuscript we used 20 co-regulated genes and included a “predicted co-regulator”, if the gene was found in correlation with another co-regulator above four times for the Q_0_/Q_30_-based network and above six times for the Q_0_/Q_56_-based network, which generally is better connected. Moreover we varied this parameter and the initial number of included hits to obtain information on the significance of this analysis.

### Colony size analysis

Colony sizes of yeast strains after transformation with polyQ proteins were documented by taking photographs of whole petri dishes. A Canon EOS 60D digital camera mounted on a repro stand was used for this purpose.

### *In vivo* NMR

^31^P-NMR was used to analyze phosphate levels *in vivo*. To this end yeast cells were washed off plates. We utilized cells directly derived from the transformation plates, where Q_0_-YFP and Q_30_-YFP could be obtained at very similar cell mass, while Q_56_-YFP expressing cells were harvested from several plates due to the toxicity of the transformation construct. Yeast cells were washed three times with 40 mM HEPES/KOH pH 7.0, 150 mM KCl to remove extracellular phosphate, which can originate directly from the agar plates. The cells were resuspended in 40 mM HEPES/KOH pH 7.0, 150 mM KCl to obtain OD_595_-levels of 300. The suspensions were then transferred to 5 mm NMR-tubes and pure D_2_O was added to 10 % final concentration in a final volume of 0.55 ml. ^31^P-NMR spectra were then measured at 25 °C using an AVANCE-III 500 NMR instrument (Bruker, Rheinstetten, Germany) equipped with a QNP cryo probe optimized for ^31^P-detection. Typically, 256 scans were assembled with a repetition time of 5 s. Spectra processing and analysis was performed with the software MestReNova 8.1. Prior to Fourier-transformation, the FID was multiplied with an exponential function (lb = 5). The spectra were calibrated to 0 ppm for external 85 % phosphoric acid.

Glucose metabolism was analyzed by ^13^C-NMR at 25 °C using a highly sensitive QNP cryo probe in the same NMR instrumentation. For this purpose, 20 mg/ml [U-^13^C_6_] glucose were added to the yeast cell suspensions and ^1^H-decoupled ^13^C NMR spectra were measured at intervals (0, 0.5, 1, 1.5, 2, 2.5 and 3 h after addition of the glucose tracer) using 256 scans and a repetition time of 5 s. Spectra were processed using the software MestReNova 8.1 using an exponential window function (lb = 1). Peak intensities were determined and the turnover rates were monitored on the basis of the glucose signal at 75.8 ppm and the ethanol signal intensity at 16.7 ppm. The kinetics of pyruvate accumulation were monitored based on the peak at 169.9 ppm and carbonate production based on the peak at 160.3 ppm.

### Analysis of intracellular iron levels

Yeast cells were washed off plates and washed in HEPES buffer as described above. The cells were resuspended in pre-warmed 5 ml 40 mM HEPES/KOH pH 7.0, 150 mM KCl supplemented with 10 mM DETAPAC, pH 7.0, and 20 mM DFO, pH 8.0, and incubated at 30 °C for 15 min. DETAPAC blocks iron import, while DFO diffuses into cells and binds unincorporated free iron in an EPR-visible ferric form [30]. Cells were then centrifuged at 4 °C, washed in ice-cold 40 mM HEPES/KOH pH 7.0, 150 mM KCl, and centrifuged again to pellet. Cell pellets were resuspended in a final volume of 300 μl of 40 mM HEPES/KOH pH 7.0, 150 mM KCl, 10 % glycerol, to gain final OD_595_-levels of 250–500. 200 μl were then transferred to a 4 mm quartz EPR tube, frozen on dry ice, and stored at −80 °C until assayed. EPR signals were measured with a Jeol JES FA 200 instrument. The spectrometer settings were as follows: temperature, −125 °C; microwave power, 10 mW; field center, 152 mT; field sweep, 100 mT; modulation amplitude, 0.25 mT; receiver gain, 1200; time constant, 0.3 s. A dewar in which samples were stored in liquid nitrogen before measurement was used to ensure constant temperature from sample to sample. Ferric chloride standards were prepared with DFO and EPR measurements were conducted as described above. The measured EPR signals were normalized according to cell density. Based on the spectra obtained from three independent samples we calculated the relative concentrations of free iron in the yeast cells.

### Analysis of aconitase activity

Yeast cells were washed off plates as described in the EPR experiments. Aconitase activity was determined using a commercially available aconitase activity assay (Sigma-Aldrich, St. Louis, MO, USA). Cells were resuspended in aconitase assay buffer and disrupted. Mechanical disruption of 950 μl of cell suspension was achieved by adding 900 mg of 0.25–0.50 mm glass beads (Carl Roth, Karlsruhe, Germany) and subsequent shaking at 4 °C in 4 pulses of 2 min at 30 Hz in a MM400 bead mill (Retsch, Haan, Germany). Cell debris was removed by centrifugation (13300 rpm, 4 °C, 10 min) and yeast lysate was further treated according to the protocol. Isocitrate was processed in the assay to yield a colorimetrically detectable product at 450 nm and compared to a measured isocitrate standard curve. The aconitase activity was determined using the equation:

$$ Aconitase\  activity\left[\frac{mU}{mL}\right]=\frac{B\times SDF}{T\times V} $$

B [nmol], amount of isocitrate generated; SDF, sample dilution factor; T [min], time reaction incubated in minutes; V [ml], sample volume. The measured aconitase activities were normalized to the protein concentration determined by Bradford assay.

### ATP-measurements

The ATP-levels were determined as described before [66, 67]. The protocol was slightly modified as harsher disruption methods had to be applied. In short: yeast cells were transformed, plated and incubated as described above. To enable analysis of the dominant colony population, large colonies were removed from pQ56 transformed plates. Plates were washed with 50 mM HEPES/KOH pH 7.8, 4 mM MgSO_4_ normalized to OD 5 and heated for 4 min at 95 °C. Mechanical disruption of 950 μl of cell suspension was achieved by adding 900 mg of 0.25–0.50 mm glass beads (Carl Roth, Karlsruhe, Germany) and subsequent shaking at 4 °C in 4 pulses of 2 min at 30 Hz in a MM400 bead mill (Retsch, Haan, Germany). After cell disruption the samples were transferred to ice and the total ATP content was analyzed by measuring the luciferase activity at 20 °C. 60 μl of the sample were added to 60 μl of a buffer containing 100 nM luciferase, 70 μM luciferin, 0.05 mg/ml BSA, 100 mM potassium phosphate buffer, pH 7.8, 25 mM glycylglycine, 0.02 mM EDTA, and bioluminescence was detected in a Tecan GENios™ microplate reader (Tecan Group, Männedorf, Switzerland).

### Determination of total NAD^+^/NADH levels

Yeast cells were washed off plates with PBS as described above. *NAD*^*+*^*/NADH levels* were determined using a commercial available NAD/NADH Quantification Kit (Sigma-Aldrich, St. Louis, MO, USA). Briefly, cells were resuspended in NAD/NADH extraction buffer and the OD_595_-levels were determined before disrupting the cells using a bead mill. Cell debris was removed by centrifugation (17000 g, 4 °C) and the lysate further treated according to the protocol. The total NAD/NADH levels were quantified colorimetrically at 450 nm and calculated by using an NADH standard curve and corrected for the deployed amount of cells.

### Detecting extracellular phosphatase activity

Extracellular phosphatase activity was analyzed as described before [68]. Yeast cells were transformed, plated and incubated as described above. Plates were washed with no-phosphate buffer, spun down and washed twice with no-phosphate medium. The cells were incubated with an OD_600_ of 5 in 1 ml at 30 °C in no-phosphate medium. To determine the phosphatase activity at different time points 50 μl of the culture were added to 200 μl ***p***-nitrophenylphosphate (5.62 mg/ml in 0.1 m sodium acetate, pH 4.2) and incubated at 20 °C for 15 min. By adding 200 μl of 10 % cold TCA the reaction was stopped and 200 μl were transferred to a new tube. Subsequently 200 μl of a saturated carbonate solution were added, mixed and spun down for 10 min at 3000 rpm.

The OD_420_ of 140 μl was recorded in quarz cuvettes in a Cary 50 UV/Vis spectrometer (Varian, Palo Alto, CA, USA). The extracellular phosphatase activity is given as the normalized OD_420_/OD_600_ x 1000.

### Fluorescence microscopy

For monitoring the fluorescence of GFP fused marker proteins an Axiovert 200 microscope (Zeiss Biomedical, Oberkochen, Germany) and the appropriate filter sets for GFP, mCherry fluorescence and DIC for transmitted light were deployed. The exposure time was not changed observing pQ0 and pQ56 transformed yeasts if not stated otherwise. For signal intensity quantification the mean intensity per pixel in the GFP channel was measured in areas densely populated with cells using ImageJ. The mean background intensity was similarly determined in empty image areas and subtracted from the mean intensity of fluorescent cells. The mean fluorescence was determined in triplicates.

### Mitochondrial staining

Mitochondria were stained with MitoTracker rhodamine (Thermo Fisher, Waltham, MA, USA). Cells were washed off plates with PBS as described before and stained according to the manufacturer's protocol. In short, 250 nM MitoTracker was incubated for 15 min with pQ0 and pQ56 transformed yeast cells. Subsequently fluorescence was monitored without washing or further fixation of the cells.

### Statistical analysis

Statistical analysis was performed using a double paired t-test with the OriginPro 8.6 software. Statistical analysis of microarray data was performed in Excel using the T.TEST function on the individual data sets.

#### Availability of supporting data

The data sets supporting the results of this article are included within the article and its additional files.

## Additional files

Additional file 1:
**Comparison between averaged Q**_**0**_ **/Q**_**56**_ **-YFP expression levels of microarrays.** A) Relative expression differences with standard deviation. B) Relative expression differences with threshold levels for the inclusion in the initial hits list. C) Determination of noise levels for the exclusion of genes from the initial hits list. *S. pombe* MAS5 values from the microarray set Q0_2d. The blacsk line represents the applied noise threshold D) Correlation between Q0_3d and Q30_3d MAS5 signals. The lines represent the applied noise threshold. (PDF 144 kb) Additional file 2:
**Genes with reduced expression in Q**_**56**_ **-YFP colonies versus Q**_**0**_ **-YFP colonies.** The table summarizes the averaged expression differences from the comparison of four data sets (Q0_2d, Q0_3d, Q56_3d and Q56_4d). Each combination was evaluated and the average expression difference was obtained. Standard deviation was calculated from these values. p-values were obtained by using the t-test. Hits with a p-value greater than 0.05 are indicated in grey. (DOCX 23 kb) Additional file 3:
**Predicted co-regulated genes.** All predicted co-regulated genes integrated into the networks are listed together with their log_2_ regulation. (DOCX 19 kb) Additional file 4:
**Genes with reduced expression in Q**_**30**_ **-YFP colonies versus Q**_**0**_ **-YFP colonies.** The table summarizes the expression differences of two data sets (Q0_3d, Q30_3d). Standard deviation and p-values were obtained as described above. Hits with a p-value greater than 0.05 are indicated in grey. (DOCX 16 kb) Additional file 5:
**Reduced expression between Q**_**30**_ **-YFP and Q**_**0**_ **-YFP yeast cells.** A) Network of genes, which are lower expressed in Q_30_-YFP producing cells compared to Q_0_-YFP producing cells. Genes are colored in accordance to their log differences (100 % green: log_2_ > 1, 75 % green: 1 > log_2_ > 0.75, 50 % green: 0.75 > log_2_ > 0.5, 25 % green: 0.5 > log_2_ > 0.25, white: 0.25 > log_2_ > -0.25, 25 % red: -0.25 > log_2_ > -0.5, 50 % red: -0.5 > log_2_ > -0.75, 75 % red: -0.75 > log_2_ > -1, 100 % green: -1 > log_2_). Pink frames highlight the predicted co-regulated genes. B) Statistical analysis of predicted co-regulators within Figure S2A. The line shows the number of genes within the respective category, while the vertical bar chart indicates the percentage of these genes predicted by the SPELL-correlations. (PDF 180 kb) Additional file 6:
**Expression differences between Q**_**56**_ **-YFP and Q**_**30**_ **-YFP yeast cells.** Cluster regulation in four possible combinations of the Q_0_/Q_56_ and Q_0_/Q_30_ experimental data sets. A) Consistency of the down-regulation of the genes in phosphate uptake and transport. Each cluster represents a comparison of different Q_0_ and Q_30_ (lower row) and Q_56_ experiments (upper row). B) Consistency of the up-regulation of the iron and the sulfur cluster. Each cluster represents a comparison of different Q_0_ and Q_30_ (lower row) and Q_56_ experiments (upper row). (PDF 4826 kb) Additional file 7:
**Peak assignments in the** ^**31**^ **P-NMR spectra.** Peaks as observed in the ^31^P NMR spectra were assigned with the help of databases and literature. (DOCX 14 kb) Additional file 8:
**Genes with increased expression in Q**_**56**_ **-YFP colonies versus Q**_0_ **-YFP colonies.** The table summarizes the averaged expression differences from the comparison of four data sets (Q0_2d, Q0_3d, Q56_3d and Q56_4d). Each combination was evaluated and the average expression difference was obtained. Standard deviation was calculated from these values. p-values were obtained by using the t-test. (DOCX 22 kb) Additional file 9:
**Genes with unaltered expression between Q**_**56**_ **-YFP and Q**_**0**_ **-YFP yeast cells.** Network of genes regulated in Q_56_-YFP expressing cells compared to Q_0_-YFP expressing cells using the same color code as Fig. **2**a and **4**a. Genes are colored accordance to their log differences (100 % green: log_2_ > 2, 75 % green: 2 > log_2_ > 1.5, 50 % green: 1.5 > log_2_ > 1, 25 % green: 1 > log_2_ > 0.5, white: 0.5 > log_2_ > -0.5, 25 % red: -0.5 > log_2_ > -1, 50 % red: -1 > log_2_ > -1.5, 75 % red: -1.5 > log_2_ > -2, 100 % green: -2 > log_2_). Genes are clustered according to their GO-Terms. (PDF 307 kb) Additional file 10:
**Genes with increased expression in Q**_**30**_ **-YFP colonies versus Q**_**0**_ **-YFP colonies.** The table summarizes the expression differences of two data sets (Q0_3d, Q30_3d). Standard deviation and p-values were obtained as described above. (DOCX 16 kb) Additional file 11:
**Enhanced expression between Q**_**30**_ **-YFP and Q**_**0**_ **-YFP yeast cells.** A) Network of genes, which are higher expressed in Q_30_-YFP expressing cells compared to Q_0_-YFP expressing cells. Genes are colored in accordance to their log differences (100 % green: log_2_ > 1, 75 % green: 1 > log_2_ > 0.75, 50 % green: 0.75 > log_2_ > 0.5, 25 % green: 0.5 > log_2_ > 0.25, white: 0.25 > log_2_ > -0.25, 25 % red: -0.25 > log_2_ > -0.5, 50 % red: -0.5 > log_2_ > -0.75, 75 % red: -0.75 > log_2_ > -1, 100 % green: -1 > log_2_). Blue frames highlight the predicted co-regulated genes. B) Statistical analysis of predicted co-regulators within Additional file 6A. The line shows the number of genes within the respective category, while the vertical bar chart indicates the percentage of these genes predicted by the SPELL-correlations. (PDF 1709 kb) Additional file 12:
**EPR-measurement of yeast cells**. A representative EPR measurement is shown. Detection of iron-content in Q_0_-YFP, Q_30_-YFP and Q_56_-YFP producing yeast cells. For detailed information see methods section. (PDF 363 kb) Additional file 13:
**Peak assignments in the** ^**13**^ **C-NMR spectra.** Peaks as observed in the ^13^C NMR spectra were assigned with the help of databases and literature. (DOCX 14 kb) Additional file 14:
**Metabolism of [U-** ^**13**^ **C**_**6**_ **]-glucose in polyQ intoxicated yeast monitored by** ^**13**^ **C-NMR.** A) NMR-analysis of [U-^13^C_6_]-glucose in the absence of yeast. B) NMR-analysis of [U-^13^C_6_]-glucose in the presence of Q_0_-YFP expressing yeasts or C) in the presence of Q_56_-YFP expressing yeasts. D) Kinetics of the metabolite production based on the peak at 124 ppm by pQ0 transformed yeasts (blue circles), pQ56 transformed yeasts (red square) or no yeasts present (black triangle). E) Kinetics of [U-^13^C_6_]-glucose consumption based on the peak at 60 ppm by pQ0 transformed yeasts (blue circles), pQ56 transformed yeasts (red square) or no yeasts present (black triangle). Without yeasts [U-^13^C_6_]-glucose is not metabolized. The chemical shift positions are assigned in Additional file 13. F) Assigned spectra in the range of -20 ppm and 230 ppm after 2 h of glucose metabolism for pQ0- and pQ56- transformed yeast cells. G) Detailed view on pyruvate (Pyr) and carbonate signals. Assigned spectra in the range of 156 ppm to 210 ppm expose the smaller peaks. Peak labeling corresponds to Additional file 13. (PDF 652 kb) Additional file 15:
**Deletion of mitochondrial fusion mediators** ***fzo1*** **Δ and** ***mgm1*** **Δ.** The deletion of mitochondrial fusion mediators *fzo1*Δ and *mgm1*Δ results in loss of colony growth upon Q_56_-YFP expression. BY4741, *﻿fzo1*Δ and *mgm1*Δ strains after transformation of either Q_0_-YFP (upper row) or Q_56_-YFP (lower row). The respective deletion is indicated on the top. Growth was documented after 4 days of incubation at 30 °C. Scale bar represents 10 mm. (PDF 14378 kb) Additional file 16:
**GFP-strains used in this manuscript.** For the analysis of cellular structures the respective strains of the Yeast GFP fusion collection in the background of EY0986 (*MAT* **a**; *his3*Δ*1*; *leu2*Δ*0*; *met15*Δ*0*; *ura3*Δ*0*) were deployed and transformed with either p Q_0_ or p Q_56_ [59]. Sul1 was not investigated further as it only shows very faint fluorescence. (DOCX 14 kb)

## Abbreviations

polyQ: Stretch of polyglutamine residues
NMR: Nuclear magnetic resonance spectroscopy
EPR: Electron paramagnetic resonance spectroscopy
DFO: Desferroxiamine
DETAPAC: Diethylenetriamine pentaacetic acid
Fe-S cluster: Iron-sulfur cluster
DTT: Dithiothreitol
EDTA: Ethylenediaminetetraacetic acid
D_2_O: Deuterated water
YPD: Yeast extract peptone dextrose medium
SD media: Synthetic defined media
YFP: Yellow fluorescent protein
WT: Wild type
pica: Polyglutamine-induced cellular arrest
