# Supplementary figures and images for: Polyglutamine toxicity in yeast induces metabolic alterations and mitochondrial defects
Source: BMC Genomics. 2015 Sep 3;16(1):662. doi: 10.1186/s12864-015-1831-7 (PMC4558792; doi:10.1186/s12864-015-1831-7)

## Additional file 1

A

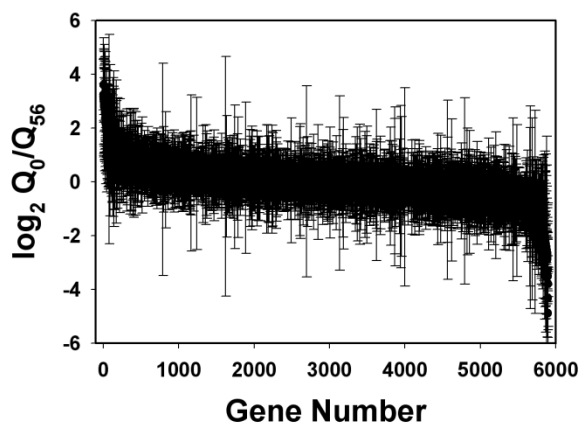

B

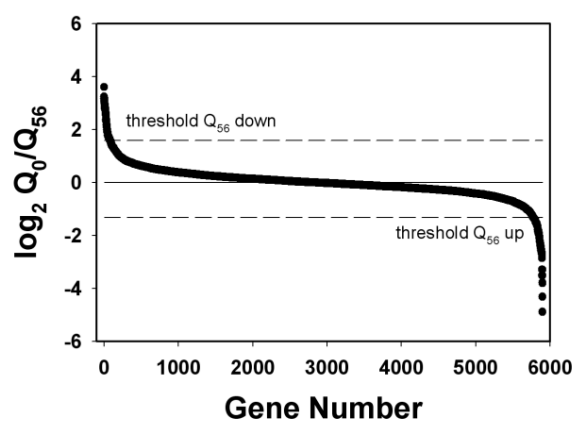

C

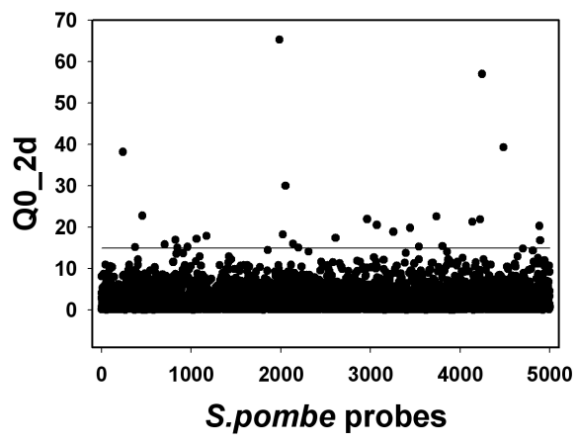

D

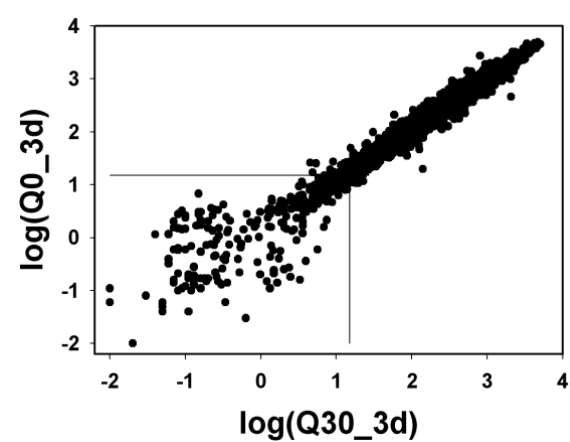

Supplement: Additional file 1: — Comparison between averaged Q 0 /Q 56 -YFP expression levels of microarrays. A) Relative expression differences with standard deviation. B) Relative expression differences with threshold levels for the inclusion in the initial hits list. C) Determination of noise levels for the exclusion of genes from the initial hits list. S. pombe MAS5 values from the microarray set Q0_2d. The blacsk line represents the applied noise threshold D) Correlation between Q0_3d and Q30_3d MAS5 signals. The lines represent the applied noise threshold. (PDF 144 kb) [file 12864_2015_1831_MOESM1_ESM.pdf]

Additional file 5

A

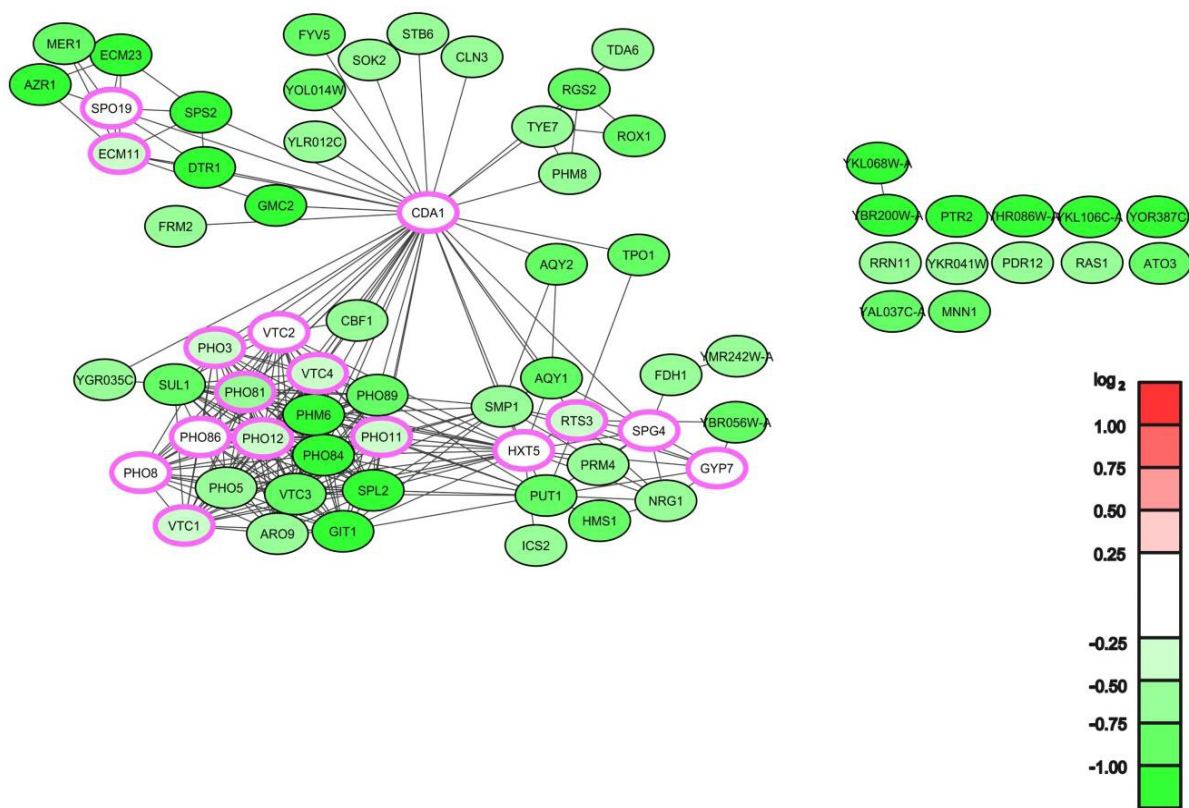

B

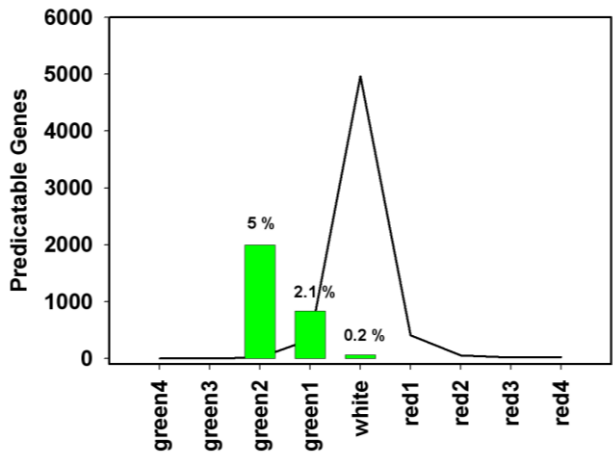

Supplement: Additional file 5: — Reduced expression between Q 30 -YFP and Q 0 -YFP yeast cells. A) Network of genes, which are lower expressed in Q30-YFP producing cells compared to Q0-YFP producing cells. Genes are colored in accordance to their log differences (100 % green: log2 > 1, 75 % green: 1 > log2 > 0.75, 50 % green: 0.75 > log2 > 0.5, 25 % green: 0.5 > log2 > 0.25, white: 0.25 > log2 > -0.25, 25 % red: -0.25 > log2 > -0.5, 50 % red: -0.5 > log2 > -0.75, 75 % red: -0.75 > log2 > -1, 100 % green: -1 > log2). Pink frames highlight the predicted co-regulated genes. B) Statistical analysis of predicted co-regulators within Figure S2A. The line shows the number of genes within the respective category, while the vertical bar chart indicates the percentage of these genes predicted by the SPELL-correlations. (PDF 180 kb) [file 12864_2015_1831_MOESM5_ESM.pdf]

A

A

1

2

3

4

Phosphate cluster

Q56

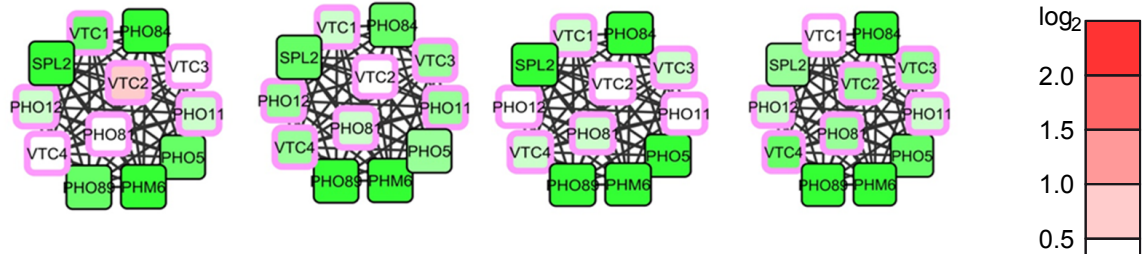

Q30

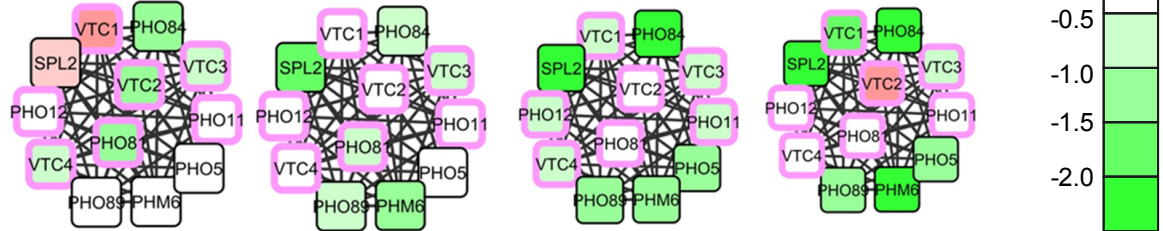

1

2

3

4

B

Iron cluster

Q56

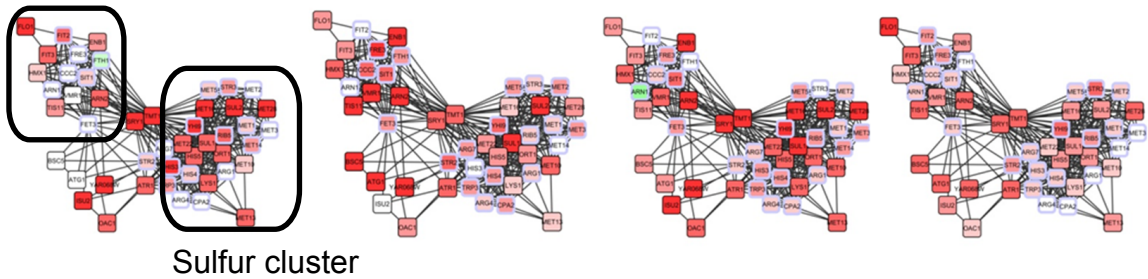

Iron cluster

Q30

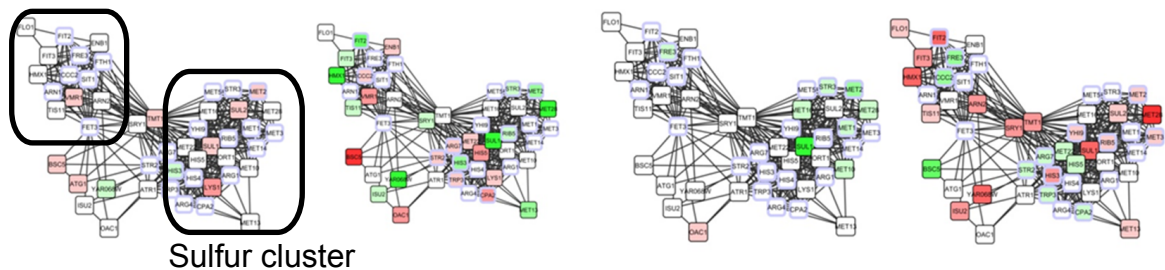

Supplement: Additional file 6: — Expression differences between Q 56 -YFP and Q 30 -YFP yeast cells. Cluster regulation in four possible combinations of the Q0/Q56 and Q0/Q30 experimental data sets. A) Consistency of the down-regulation of the genes in phosphate uptake and transport. Each cluster represents a comparison of different Q0 and Q30 (lower row) and Q56 experiments (upper row). B) Consistency of the up-regulation of the iron and the sulfur cluster. Each cluster represents a comparison of different Q0 and Q30 (lower row) and Q56 experiments (upper row). (PDF 4826 kb) [file 12864_2015_1831_MOESM6_ESM.pdf]

Additional file 9

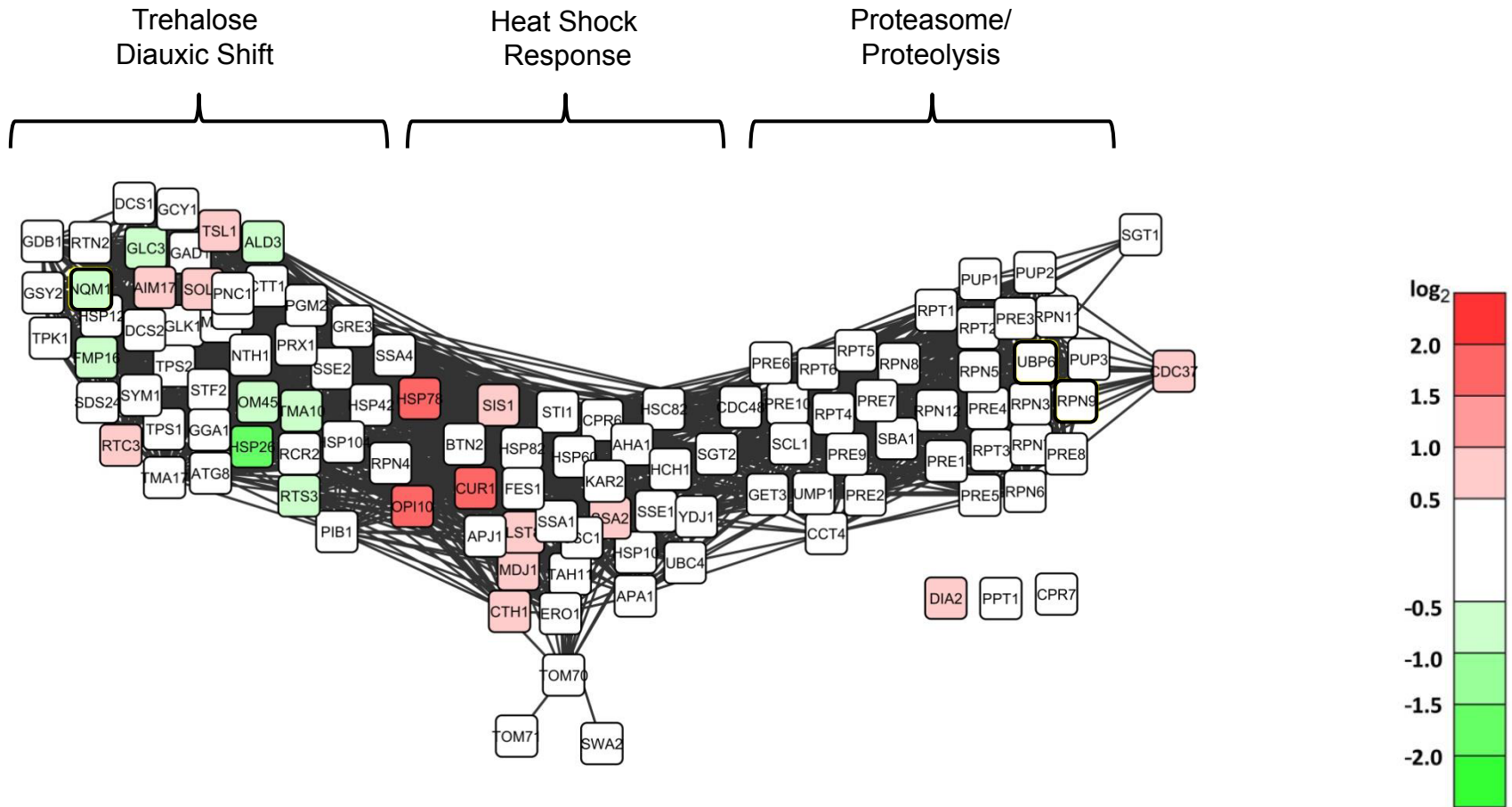

Supplement: Additional file 9: — Genes with unaltered expression between Q 56 -YFP and Q 0 -YFP yeast cells. Network of genes regulated in Q56-YFP expressing cells compared to Q0-YFP expressing cells using the same color code as Fig. 2a and 4a. Genes are colored accordance to their log differences (100 % green: log2 > 2, 75 % green: 2 > log2 > 1.5, 50 % green: 1.5 > log2 > 1, 25 % green: 1 > log2 > 0.5, white: 0.5 > log2 > -0.5, 25 % red: -0.5 > log2 > -1, 50 % red: -1 > log2 > -1.5, 75 % red: -1.5 > log2 > -2, 100 % green: -2 > log2). Genes are clustered according to their GO-Terms. (PDF 307 kb) [file 12864_2015_1831_MOESM9_ESM.pdf]

A

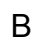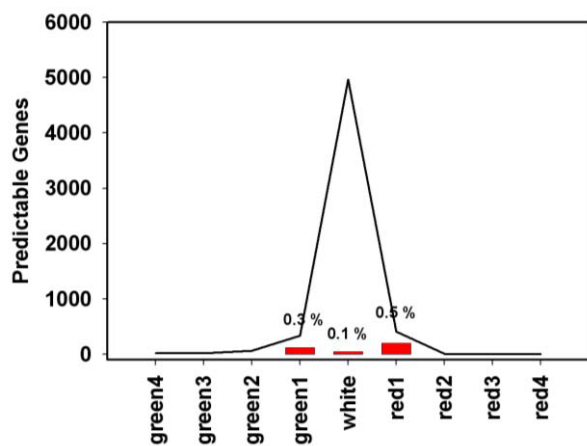

Supplement: Additional file 11: — Enhanced expression between Q 30 -YFP and Q 0 -YFP yeast cells. A) Network of genes, which are higher expressed in Q30-YFP expressing cells compared to Q0-YFP expressing cells. Genes are colored in accordance to their log differences (100 % green: log2 > 1, 75 % green: 1 > log2 > 0.75, 50 % green: 0.75 > log2 > 0.5, 25 % green: 0.5 > log2 > 0.25, white: 0.25 > log2 > -0.25, 25 % red: -0.25 > log2 > -0.5, 50 % red: -0.5 > log2 > -0.75, 75 % red: -0.75 > log2 > -1, 100 % green: -1 > log2). Blue frames highlight the predicted co-regulated genes. B) Statistical analysis of predicted co-regulators within Additional file 6A. The line shows the number of genes within the respective category, while the vertical bar chart indicates the percentage of these genes predicted by the SPELL-correlations. (PDF 1709 kb) [file 12864_2015_1831_MOESM11_ESM.pdf]

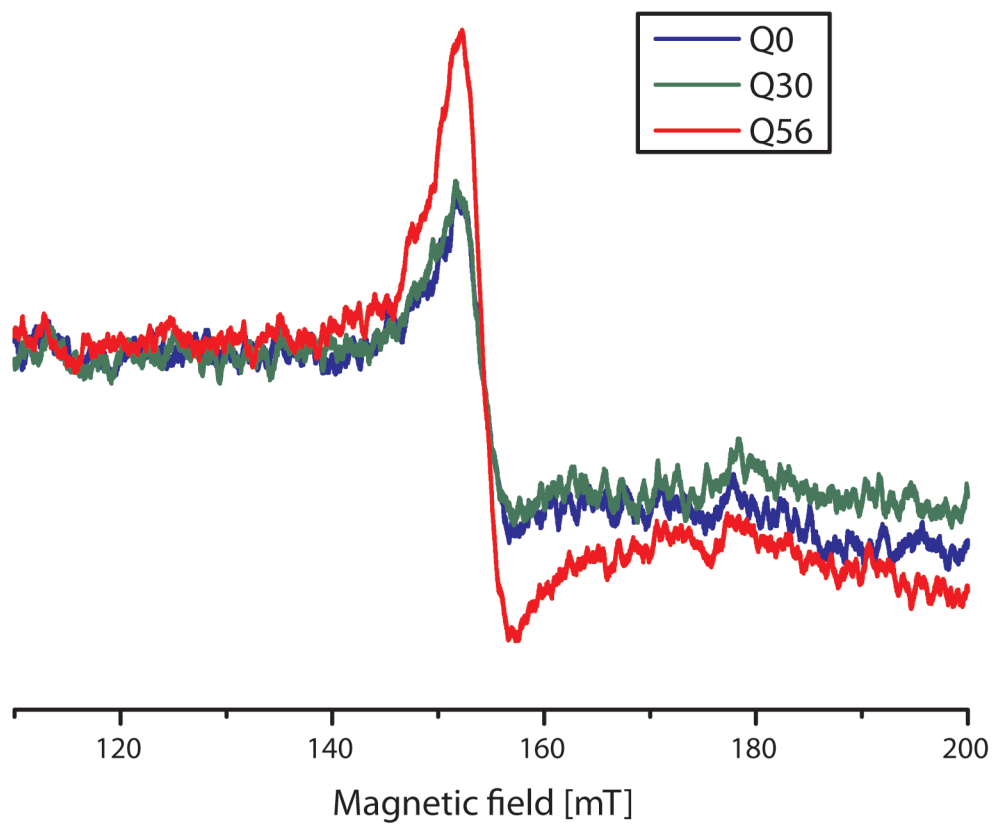

Supplement: Additional file 12: — EPR-measurement of yeast cells. A representative EPR measurement is shown. Detection of iron-content in Q0-YFP, Q30-YFP and Q56-YFP producing yeast cells. For detailed information see methods section. (PDF 363 kb) [file 12864_2015_1831_MOESM12_ESM.pdf]

A    Glucose alone    0h

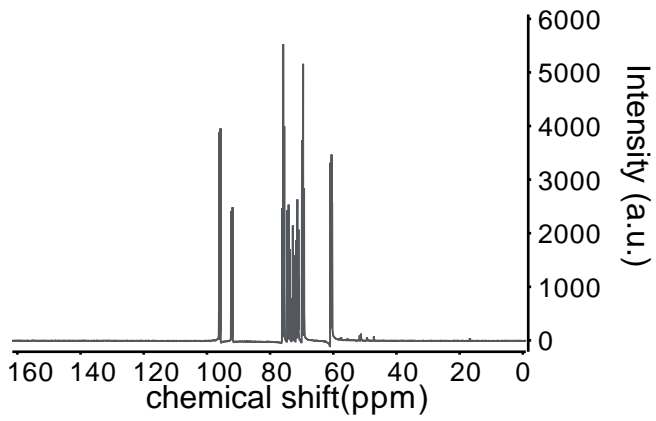

after 2h

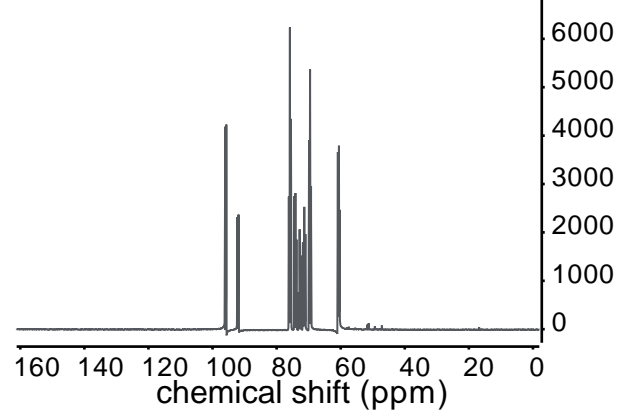

B Q0 0h

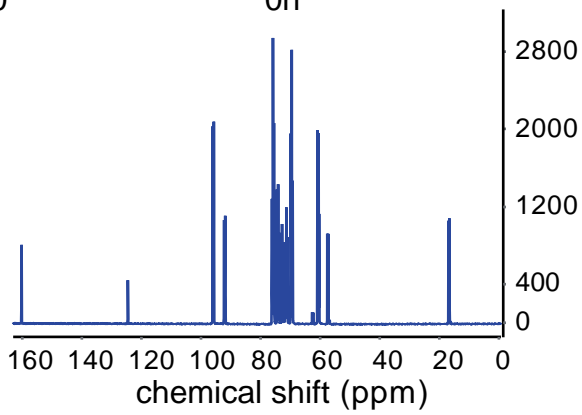

after 2h

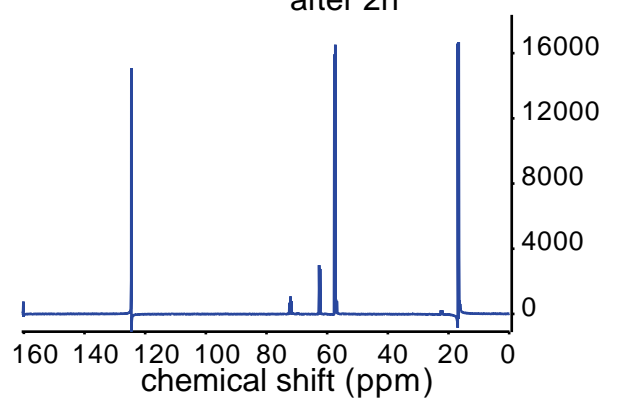

C Q56 0h

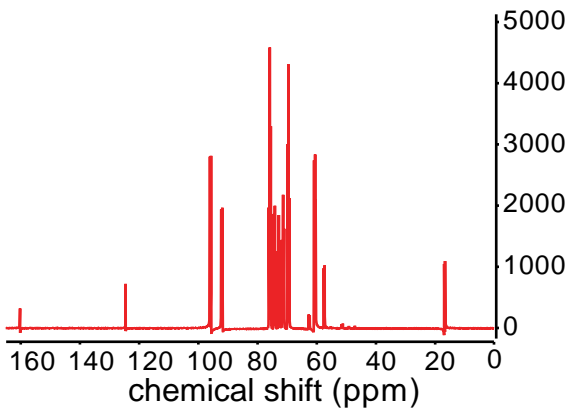

after 2h

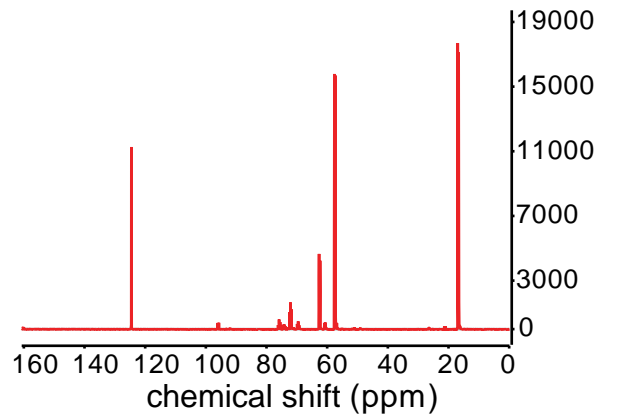

D

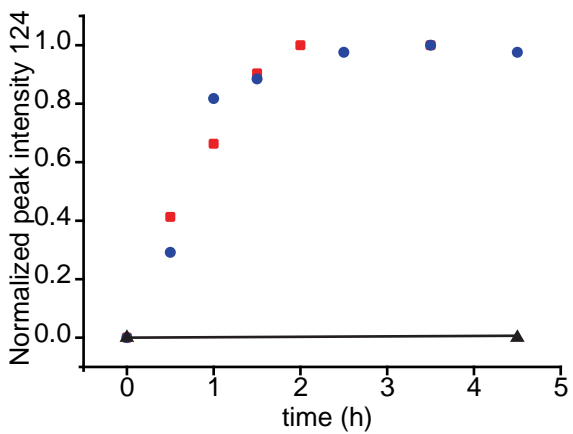

E

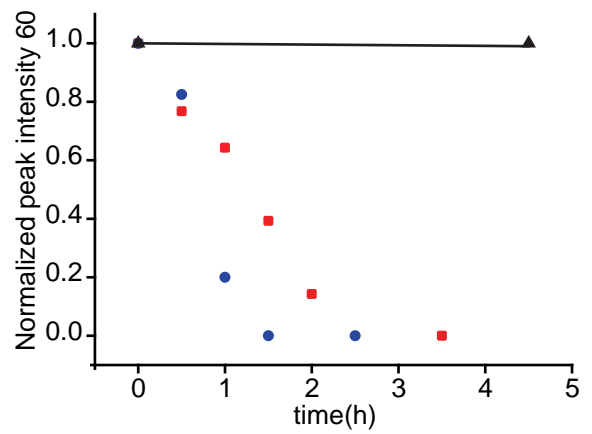

F

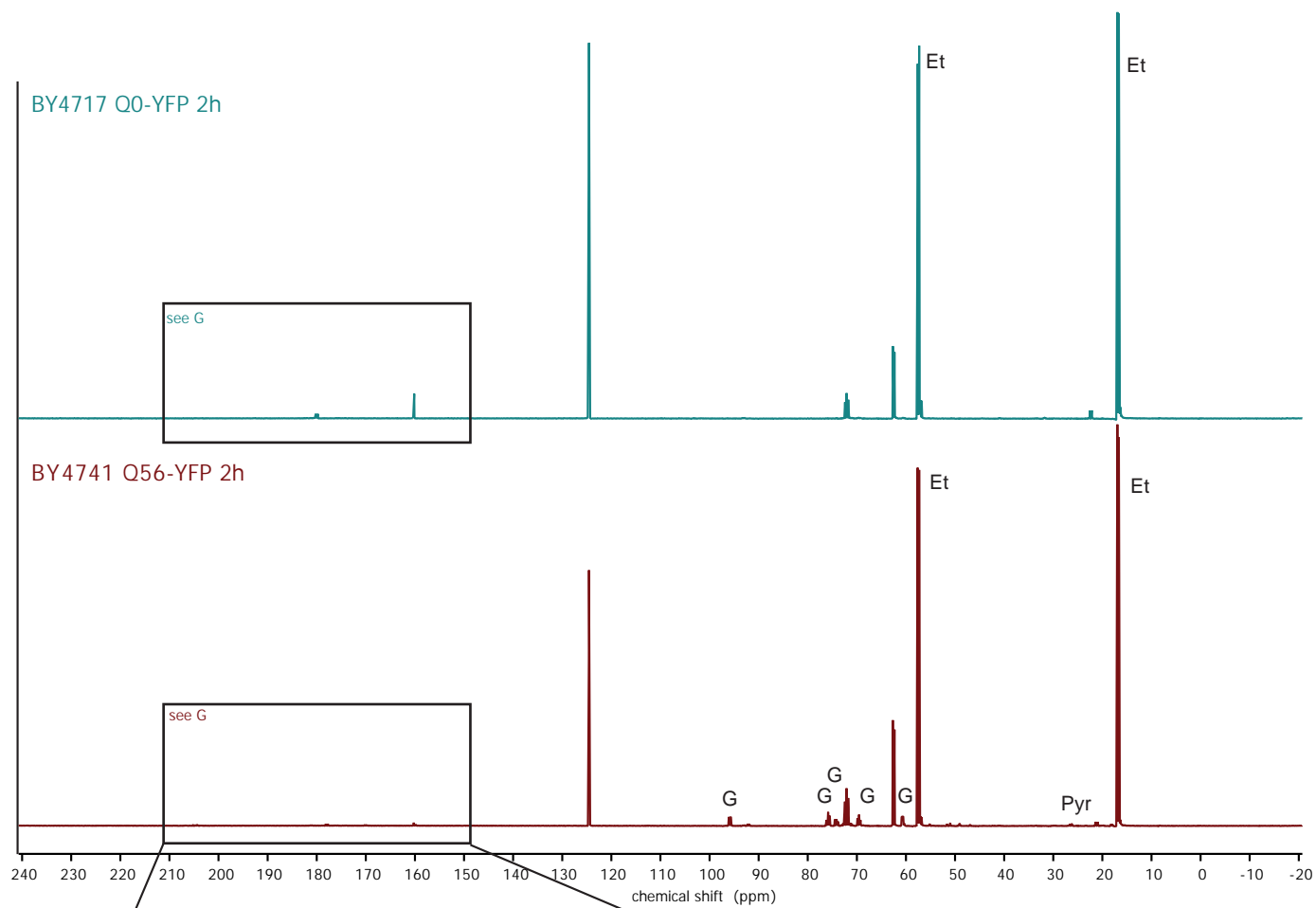

G

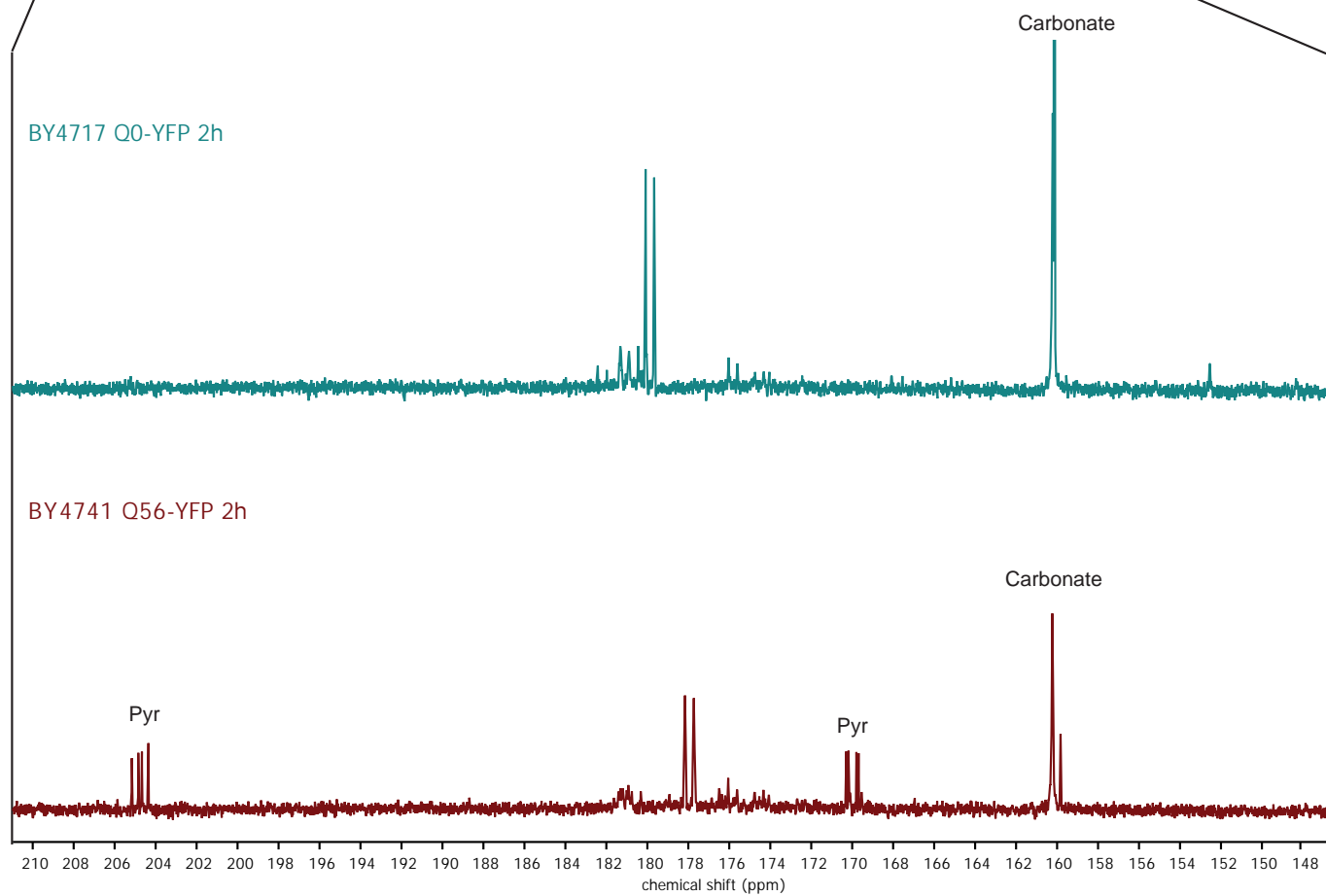

Supplement: Additional file 14: — Metabolism of [U- 13 C 6 ]-glucose in polyQ intoxicated yeast monitored by 13 C-NMR. A) NMR-analysis of [U-13C6]-glucose in the absence of yeast. B) NMR-analysis of [U-13C6]-glucose in the presence of Q0-YFP expressing yeasts or C) in the presence of Q56-YFP expressing yeasts. D) Kinetics of the metabolite production based on the peak at 124 ppm by pQ0 transformed yeasts (blue circles), pQ56 transformed yeasts (red square) or no yeasts present (black triangle). E) Kinetics of [U-13C6]-glucose consumption based on the peak at 60 ppm by pQ0 transformed yeasts (blue circles), pQ56 transformed yeasts (red square) or no yeasts present (black triangle). Without yeasts [U-13C6]-glucose is not metabolized. The chemical shift positions are assigned in Additional file 13. F) Assigned spectra in the range of -20 ppm and 230 ppm after 2 h of glucose metabolism for pQ0- and pQ56- transformed yeast cells. G) Detailed view on pyruvate (Pyr) and carbonate signals. Assigned spectra in the range of 156 ppm to 210 ppm expose the smaller peaks. Peak labeling corresponds to Additional file 13. (PDF 652 kb) [file 12864_2015_1831_MOESM14_ESM.pdf]

Additional file 15

BY4717

*fzo1* $\Delta$

*mgm1* $\Delta$

pQ0

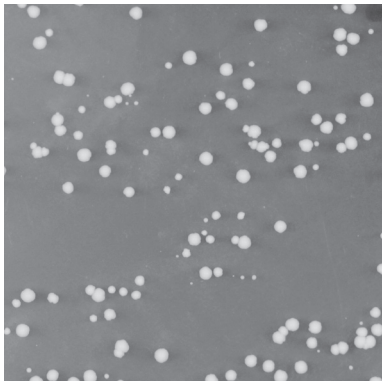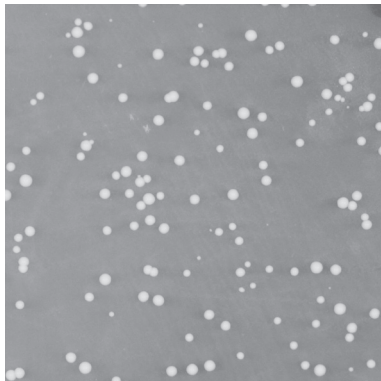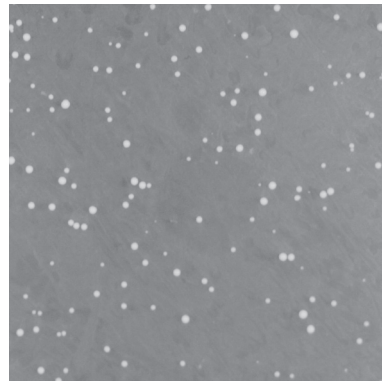

pQ56

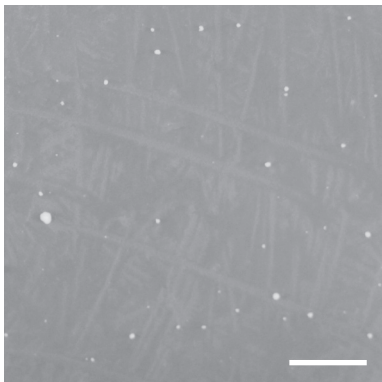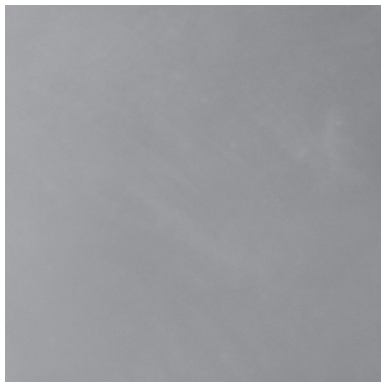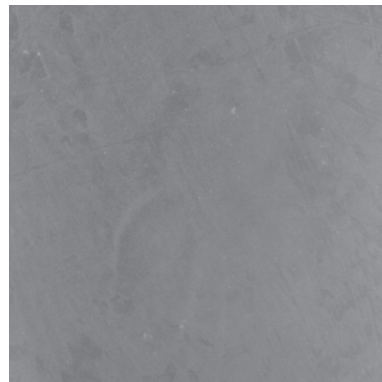

Supplement: Additional file 15: — Deletion of mitochondrial fusion mediators fzo1 Δ and mgm1 Δ. The deletion of mitochondrial fusion mediators fzo1Δ and mgm1Δ results in loss of colony growth upon Q56-YFP expression. BY4741, ﻿fzo1Δ and mgm1Δ strains after transformation of either Q0-YFP (upper row) or Q56-YFP (lower row). The respective deletion is indicated on the top. Growth was documented after 4 days of incubation at 30 °C. Scale bar represents 10 mm. (PDF 14378 kb) [file 12864_2015_1831_MOESM15_ESM.pdf]
