# Supplementary material for: Polyglutamine toxicity in yeast induces metabolic alterations and mitochondrial defects
Source: BMC Genomics. 2015 Sep 3;16(1):662. doi: 10.1186/s12864-015-1831-7 (PMC4558792; doi:10.1186/s12864-015-1831-7)
Supplement: Additional file 2: — Genes with reduced expression in Q 56 -YFP colonies versus Q 0 -YFP colonies. The table summarizes the averaged expression differences from the comparison of four data sets (Q0_2d, Q0_3d, Q56_3d and Q56_4d). Each combination was evaluated and the average expression difference was obtained. Standard deviation was calculated from these values. p-values were obtained by using the t-test. Hits with a p-value greater than 0.05 are indicated in grey. (DOCX 23 kb) [file 12864_2015_1831_MOESM2_ESM.docx]

**Additional file 2: Genes with reduced expression in Q_56_-YFP colonies versus Q_0_-YFP colonies.**

| **ID** | **Gene Symbol** | **log_2_ Q_0_/Q_56_** | **Standard**  **deviation** | **p-value** |
| --- | --- | --- | --- | --- |
| YPR192W | AQY1 | 3.61 | 0.93 | 0.00081737 |
| YCR010C | ADY2 | 3.24 | 1.02 | 0.0290809 |
| YLR377C | FBP1 | 3.17 | 2.18 | 0.08286075 |
| YNR056C | BIO5 | 3.08 | 0.58 | 0.00470883 |
| YNL117W | MLS1 | 2.99 | 1.60 | 0.0470131 |
| YGL158W | RCK1 | 2.98 | 0.69 | 0.01950588 |
| YHR139C | SPS100 | 2.97 | 1.91 | 0.04871947 |
| YMR323W, YOR393W, YPL281C | ERR1, ERR2, ERR3 | 2.95 | 2.01 | 0.05846673 |
| YDR281C | PHM6 | 2.93 | 0.70 | 0.00069456 |
| YOR032C | HMS1 | 2.87 | 1.00 | 0.02086733 |
| YPL223C | GRE1 | 2.83 | 2.01 | 0.04599407 |
| YJL045W | --- | 2.83 | 1.51 | 0.03996189 |
| YGR236C | SPG1 | 2.81 | 1.69 | 0.04981097 |
| YKR097W | PCK1 | 2.80 | 1.59 | 0.04787258 |
| YOR388C, YPL275W | FDH1, FDH2 | 2.79 | 0.86 | 0.00062556 |
| YAL062W | GDH3 | 2.71 | 1.95 | 0.06091858 |
| YMR175W | SIP18 | 2.68 | 1.83 | 0.04602545 |
| YCR021C | HSP30 | 2.62 | 2.04 | 0.08092449 |
| YIL057C | RGI2 | 2.61 | 1.04 | 0.04984244 |
| YOR107W | RGS2 | 2.54 | 1.57 | 0.04738938 |
| YBR296C | PHO89 | 2.45 | 0.81 | 0.0443598 |
| YDR536W | STL1 | 2.39 | 2.07 | 0.15249177 |
| YGR052W | FMP48 | 2.37 | 0.21 | 0.00832945 |
| YBR066C | NRG2 | 2.33 | 0.32 | 0.00159371 |
| YNR002C | ATO2 | 2.30 | 0.80 | 0.0017669 |
| YBR157C | ICS2 | 2.24 | 0.51 | 0.01929475 |
| YBR050C | REG2 | 2.19 | 1.25 | 0.09672312 |
| YOL152W | FRE7 | 2.19 | 1.06 | 0.02945964 |
| YML123C | PHO84 | 2.17 | 0.33 | 0.01136453 |
| YOR178C | GAC1 | 2.13 | 1.44 | 0.0965377 |
| YMR081C | ISF1 | 2.11 | 1.66 | 0.11743186 |
| YBL075C | SSA3 | 2.04 | 0.54 | 0.02274132 |
| YGR256W | GND2 | 1.99 | 1.42 | 0.03496092 |
| YHR136C | SPL2 | 1.99 | 0.68 | 0.00472149 |
| YPR065W | ROX1 | 1.98 | 0.92 | 0.00723834 |
| YCL036W | GFD2 | 1.93 | 0.86 | 0.00410337 |
| YHR160C | PEX18 | 1.91 | 1.01 | 0.05309219 |
| YNL142W | MEP2 | 1.90 | 1.05 | 0.06594156 |
| YLR142W | PUT1 | 1.89 | 1.52 | 0.13610642 |
| YNR058W | BIO3 | 1.88 | 0.73 | 0.02455291 |
| YNR057C | BIO4 | 1.85 | 1.05 | 0.02461184 |
| YNL269W | BSC4 | 1.81 | 1.53 | 0.11786821 |
| YBR093C | PHO5 | 1.79 | 0.33 | 0.02237015 |
| YIL160C | POT1 | 1.78 | 0.78 | 0.02638647 |
| YKL107W | --- | 1.77 | 1.14 | 0.06675667 |
| YGL162W | SUT1 | 1.76 | 0.48 | 0.02785988 |
| YJR115W | --- | 1.76 | 0.85 | 0.01625853 |
| YBR072W | HSP26 | 1.74 | 1.66 | 0.08631927 |
| YKR093W | PTR2 | 1.73 | 0.96 | 0.11303896 |
| YKL043W | PHD1 | 1.73 | 0.34 | 0.00219033 |
| YPL201C | YIG1 | 1.72 | 1.90 | 0.25307398 |
| YNR014W | --- | 1.71 | 1.73 | 0.04996757 |
| YLR053C | --- | 1.70 | 1.00 | 0.07572893 |
| YOR376W-A | --- | 1.70 | 0.86 | 0.10732706 |
| YPL054W | LEE1 | 1.69 | 1.42 | 0.10407407 |
| YGR201C | --- | 1.68 | 1.29 | 0.04786668 |
| YPL057C | SUR1 | 1.68 | 0.69 | 0.031226 |
| YGR121C | MEP1 | 1.68 | 0.71 | 0.01771003 |
| YDL218W | --- | 1.68 | 0.84 | 0.04976561 |
| YHR022C | --- | 1.68 | 0.25 | 0.005146 |
| YJR108W | ABM1 | 1.68 | 1.71 | 0.07610121 |
| YJR095W | SFC1 | 1.67 | 1.93 | 0.23675555 |
| YKL093W | MBR1 | 1.66 | 0.95 | 0.04478269 |
| YMR303C | ADH2 | 1.65 | 0.49 | 0.0400323 |
| YNL194C | --- | 1.65 | 0.87 | 0.10056003 |
| YOR028C | CIN5 | 1.63 | 0.66 | 0.00347329 |
| YMR175W-A | --- | 1.61 | 1.79 | 0.13563467 |
| YOL084W | PHM7 | 1.59 | 0.29 | 0.01975799 |
| YER150W | SPI1 | 1.59 | 0.68 | 0.02702445 |
| YHR096C | HXT5 | 1.59 | 1.02 | 0.08141928 |
| YPR013C | --- | 1.58 | 0.54 | 0.02577641 |
| YGR249W | MGA1 | 1.58 | 0.36 | 0.02992433 |
| YER065C | ICL1 | 1.58 | 1.62 | 0.2384934 |
| YEL019C | MMS21 | 1.57 | 1.10 | 0.05747169 |
| YHL024W | RIM4 | 1.57 | 0.52 | 0.07000804 |
| YMR280C | CAT8 | 1.57 | 0.99 | 0.15233503 |
