# Supplementary material for: Polyglutamine toxicity in yeast induces metabolic alterations and mitochondrial defects
Source: BMC Genomics. 2015 Sep 3;16(1):662. doi: 10.1186/s12864-015-1831-7 (PMC4558792; doi:10.1186/s12864-015-1831-7)
Supplement: Additional file 3: — Predicted co-regulated genes. All predicted co-regulated genes integrated into the networks are listed together with their log2 regulation. (DOCX 19 kb) [file 12864_2015_1831_MOESM3_ESM.docx]

**Additional file 3: Predicted co-regulated genes.**

| **ID** | **log_2_ Q_56_/Q_0_**  upregulated |
| --- | --- |
| FMP23 | 1.302 |
| MCH4 | 1.259 |
| RIB5 | 1.241 |
| SNZ1 | 1.227 |
| SIT1 | 1.178 |
| FRE3 | 1.171 |
| STR2 | 1.142 |
| ADH5 | 1.128 |
| MET5 | 1.11 |
| TRP3 | 1.093 |
| HIS3 | 1.067 |
| PCL5 | 1.055 |
| FET3 | 0.93 |
| STR3 | 0.883 |
| HIS4 | 0.857 |
| MET1 | 0.83 |
| ARG7 | 0.8 |
| HOM3 | 0.793 |
| MET3 | 0.763 |
| MET2 | 0.754 |
| ARO3 | 0.748 |
| FIT2 | 0.727 |
| CCC2 | 0.7 |
| CPA2 | 0.661 |
| GAS4 | 0.565 |
| ARG1 | 0.55 |
| MET14 | 0.458 |
| ARG4 | 0.424 |
| FTH1 | 0.345 |
| ARN1 | 0.221 |
| TEP1 | 0.109 |
| YAT2 | -0.04 |
| CDA1 | -0.79 |
|  |  |
|  |  |
|  |  |
|  |  |
|  |  |
|  |  |
|  |  |
|  |  |
|  |  |
|  |  |
|  |  |
|  |  |
|  |  |
|  |  |
|  |  |
|  |  |
|  |  |
|  |  |
|  |  |
|  |  |

| **ID** | **log_2_ Q_56_/Q_0_**  downregulated |
| --- | --- |
| RTC3 | -0.68 |
| SOL4 | -0.571 |
| SSE2 | -0.36 |
| HSP42 | -0.311 |
| DCS2 | -0.149 |
| TPS2 | -0.099 |
| RTN2 | -0.097 |
| UGX2 | -0.053 |
| CAT2 | -0.029 |
| CIT3 | -0.0248 |
| ATH1 | -0.004 |
| YAT2 | 0.04 |
| CTT1 | 0.095 |
| MSC1 | 0.118 |
| TFS1 | 0.184 |
| VTC2 | 0.269 |
| SPG4 | 0.271 |
| GAD1 | 0.276 |
| DDR2 | 0.316 |
| CSR2 | 0.347 |
| USV1 | 0.38 |
| NDE2 | 0.408 |
| GLC3 | 0.484 |
| FMP46 | 0.501 |
| CRC1 | 0.525 |
| VTC1 | 0.579 |
| TMA10 | 0.597 |
| NQM1 | 0.615 |
| PHO12 | 0.701 |
| PHO11 | 0.701 |
| FMP16 | 0.712 |
| POX1 | 0.76 |
| XBP1 | 0.766 |
| PHO81 | 0.777 |
| VTC4 | 0.791 |
| UIP4 | 0.797 |
| OM14 | 0.809 |
| VTC3 | 0.898 |
| ALD3 | 0.922 |
| OM45 | 0.957 |
| FOX2 | 0.965 |
| HBT1 | 1.002 |
| AGX1 | 1.167 |
| FMP45 | 1.179 |
| TKL2 | 1.218 |
| ACS1 | 1.225 |
| PAI3 | 1.238 |
| CTA1 | 1.246 |
| IDP2 | 1.398 |
| FMP43 | 1.399 |
| GPX1 | 1.403 |
| BDH2 | 1.467 |
| JEN1 | 1.498 |

| **ID** | **log_2_ Q_30_/Q_0_**  upregulated |
| --- | --- |
| PHO81 | 0.535694 |
| VTC1 | 0.450666 |
| PHO3 | 0.432173 |
| VTC4 | 0.403767 |
| RTS3 | 0.359385 |
| ECM11 | 0.339758 |
| PHO11 | 0.25274 |
| PHO86 | 0.23381 |
| GYP7 | 0.157808 |
| CDA1 | 0.103845 |
| PHO8 | 0.0869279 |
| SPO19 | 0.0332391 |
| PHO12 | 0 |
| YNL018C | -0.00310448 |
| HXT5 | -0.00575683 |
| VTC2 | -0.0178667 |
| SPG4 | -0.151059 |
|  |  |

| **ID** | **log_2_ Q_30_/Q_0_**  downregulated |
| --- | --- |
| DSF1 | -0.282326 |
| HXT8 | -0.25331 |
| SET6 | -0.135692 |
| IMA4 | 0 |
| HXT16 | 0 |
| HXT9 | 0.0944172 |
| CDA1 | 0.103845 |
| IRC10 | 0.403472 |
