# Supplementary material for: Polyglutamine toxicity in yeast induces metabolic alterations and mitochondrial defects
Source: BMC Genomics. 2015 Sep 3;16(1):662. doi: 10.1186/s12864-015-1831-7 (PMC4558792; doi:10.1186/s12864-015-1831-7)
Supplement: Additional file 4: — Genes with reduced expression in Q 30 -YFP colonies versus Q 0 -YFP colonies. The table summarizes the expression differences of two data sets (Q0_3d, Q30_3d). Standard deviation and p-values were obtained as described above. Hits with a p-value greater than 0.05 are indicated in grey. (DOCX 16 kb) [file 12864_2015_1831_MOESM4_ESM.docx]

**Additional file 4: Genes with reduced expression in Q_30_-YFP colonies versus Q_0_-YFP colonies.**

| **ID** | **log_2_ Q_0_/Q_30_** | **Standard deviation** | **p-value** |
| --- | --- | --- | --- |
| ECM23  SPL2  YBR200W-A  GMC2  AZR1  SPS2  PHO84  YOR387C  PHM6  YKL106C-A  GIT1  DTR1  YHR086W-A  YKL068W-A  PTR2  PUT1  MNN1  HMS1  TPO1  RGS2  YAL037C-A  AQY2  PHO89  MER1  FYV5  AQY1  YOL014W  ATO3  ROX1  YBR056W-A  SUL1  VTC3  FDH1  YLR012C  RAS1  SMP1  YMR242W-A  NRG1  ICS2  PDR12  CLN3  PHO5  SOK2  TDA6  STB6  CBF1  YKR041W  YGR035C  ARO9  PRM4  TYE7  FRM2  PHM8  RRN11 | 3,18  2,25  1,92  1,86  1,85  1,76  1,60  1,31  1,28  1,26  1,25  1,24  1,07  1,02  1,01  0,92  0,92  0,91  0,88  0,88  0,87  0,86  0,85  0,83  0,81  0,81  0,79  0,78  0,77  0,76  0,75  0,75  0,74  0,72  0,72  0,70  0,70  0,69  0,69  0,68  0,68  0,67  0,67  0,66  0,66  0,65  0,64  0,63  0,62  0,62  0,62  0,62  0,61  0,61 | 0,91  2,35  2,10  2,41  1,23  3,17  0,70  0,61  1,06  2,16  0,06  1,10  0,13  0,93  0,64  0,72  2,12  0,95  0,18  1,87  1,50  0,48  0,45  1,15  0,39  2,52  0,60  2,15  0,90  0,99  2,36  0,12  0,37  2,15  0,69  1,37  0,83  0,89  1,00  2,36  0,43  0,63  0,46  3,29  0,74  0,25  1,59  2,37  0,73  0,69  0,38  1,45  1,25  1,83 | 0.026418567  0.231807111  0.249651058  0.308882926  0.107903856  0.422883644  0.088853085  0.0369409  0.233504022  0.43651541  0.02980307  0.070126223  0.027874112  0.188431398  0.216674331  0.00649255  0.298122144  0.011431749  0.0381046  0.255457034  0.132681299  0.003662152  0.220025758  0.40640567  0.015620062  0.643218806  0.002907657  0.617124265  0.014126419  0.271362965  0.637509146  0.121013273  0.237352958  0.617809922  0.364471291  0.147550308  0.399957298  0.035672172  0.035322279  0.482942372  0.252470486  0.3381454  0.006286456  0.654233706  0.000662645  0.242818638  0.603150257  0.703854391  0.067710972  0.407825284  0.020827219  0.600372719  0.133314253  0.380086099 |
