# Supplementary material for: Polyglutamine toxicity in yeast induces metabolic alterations and mitochondrial defects
Source: BMC Genomics. 2015 Sep 3;16(1):662. doi: 10.1186/s12864-015-1831-7 (PMC4558792; doi:10.1186/s12864-015-1831-7)
Supplement: Additional file 7: — Peak assignments in the 31 P-NMR spectra. Peaks as observed in the 31P NMR spectra were assigned with the help of databases and literature. (DOCX 14 kb) [file 12864_2015_1831_MOESM7_ESM.docx]

**Additional file 7: Peak assignments in the ^31^P-NMR spectra.**

| **Position (ppm)** | **Peak label** | **Attributed to** |
| --- | --- | --- |
| 2  - 5.5  - 24 | 1  2  3 | Inorganic Phosphate  Polyphosphate (end of chain)  Polyphosphate (internal) |
