# Supplementary material for: Polyglutamine toxicity in yeast induces metabolic alterations and mitochondrial defects
Source: BMC Genomics. 2015 Sep 3;16(1):662. doi: 10.1186/s12864-015-1831-7 (PMC4558792; doi:10.1186/s12864-015-1831-7)
Supplement: Additional file 8: — Genes with increased expression in Q 56 -YFP colonies versus Q 0 -YFP colonies. The table summarizes the averaged expression differences from the comparison of four data sets (Q0_2d, Q0_3d, Q56_3d and Q56_4d). Each combination was evaluated and the average expression difference was obtained. Standard deviation was calculated from these values. p-values were obtained by using the t-test. (DOCX 22 kb) [file 12864_2015_1831_MOESM8_ESM.docx]

**Additional file 8: Genes with increased expression in Q_56_-YFP colonies versus Q_0_-YFP colonies.**

| **ID** | **Gene Symbol** | **log_2_ Q_56_/Q_0_** | **Standard**  **deviation** | **p-value** |
| --- | --- | --- | --- | --- |
| YAR075W | --- | 5.94 | 2.65 | 0.0118319 |
| YFL053W | DAK2 | 4.89 | 1.71 | 0.00278943 |
| YDR042C | --- | 3.80 | 0.46 | 0.00311027 |
| YPL033C | SRL4 | 3.51 | 0.77 | 0.00062449 |
| YHR029C | YHI9 | 2.67 | 1.11 | 0.00511175 |
| YAR068W, YHR214W-A | --- | 2.65 | 0.77 | 0.01328436 |
| YKL183C-A | --- | 2.64 | 0.87 | 0.00025251 |
| YHR094C | HXT1 | 2.57 | 3.19 | 0.14289571 |
| YMR095C | SNO1 | 2.51 | 0.68 | 0.00057467 |
| YER175C | TMT1 | 2.46 | 0.63 | 0.0013498 |
| YCR045C | RRT12 | 2.36 | 1.04 | 0.02183959 |
| YOR298W | MUM3 | 2.35 | 0.32 | 0.0158274 |
| YLR092W | SUL2 | 2.35 | 0.90 | 0.0133767 |
| YKL218C | SRY1 | 2.29 | 0.67 | 0.00024098 |
| YBR294W | SUL1 | 2.25 | 1.04 | 0.04034834 |
| YCL026C-A | FRM2 | 2.22 | 0.66 | 0.01227384 |
| YHR157W | REC104 | 2.22 | 0.50 | 0.02986818 |
| YFL061W, YNL335W | DDI2, DDI3 | 2.05 | 0.78 | 0.00025123 |
| YOL158C | ENB1 | 1.99 | 0.88 | 0.06596865 |
| YAR050W | FLO1 | 1.95 | 0.79 | 0.0109145 |
| YIR017C | MET28 | 1.93 | 0.69 | 1.6798E-06 |
| YJL160C | --- | 1.92 | 0.45 | 0.0101294 |
| YOL017W | ESC8 | 1.91 | 0.02 | 0.01078285 |
| YHL047C | ARN2 | 1.89 | 0.32 | 0.02217488 |
| YGL263W | COS12 | 1.84 | 0.36 | 0.01414172 |
| YML116W | ATR1 | 1.84 | 0.02 | 0.01141218 |
| YOR226C | ISU2 | 1.84 | 1.56 | 0.02822063 |
| YJL047C-A | --- | 1.83 | 0.60 | 9.4709E-06 |
| YIL165C | NIT1 | 1.74 | 0.11 | 0.01479371 |
| YOR339C | UBC11 | 1.71 | 1.09 | 0.00519265 |
| YMR094W | CTF13 | 1.67 | 0.31 | 0.00633299 |
| YDL241W | --- | 1.65 | 0.53 | 0.00370141 |
| YGL021W | ALK1 | 1.65 | 0.15 | 0.01451989 |
| YPR167C | MET16 | 1.65 | 0.99 | 0.00475723 |
| YJL213W | --- | 1.64 | 0.40 | 0.01616031 |
| YDR345C | HXT3 | 1.64 | 1.57 | 0.06695841 |
| YOR130C | ORT1 | 1.61 | 0.26 | 0.0089673 |
| YLR136C | TIS11 | 1.59 | 0.40 | 0.05823717 |
| YCL049C | --- | 1.58 | 0.54 | 0.00032344 |
| YNR069C | BSC5 | 1.58 | 1.32 | 0.20898065 |
| YDR182W-A | --- | 1.56 | 0.61 | 0.04964433 |
| YPR158W | CUR1 | 1.55 | 0.31 | 0.00351943 |
| YOR383C | FIT3 | 1.54 | 0.21 | 0.00790573 |
| YGL180W | ATG1 | 1.54 | 0.95 | 0.1502267 |
| YLR134W | PDC5 | 1.52 | 0.47 | 0.0013838 |
| YAL064W | --- | 1.52 | 0.66 | 0.10343767 |
| YCR099C | --- | 1.52 | 1.05 | 0.1736527 |
| YOL091W | SPO21 | 1.51 | 0.14 | 0.01044166 |
| YDL085C-A | --- | 1.48 | 2.11 | 0.13607594 |
| YNR068C | --- | 1.48 | 0.72 | 0.11929763 |
| YLR348C | DIC1 | 1.48 | 1.19 | 0.02931345 |
| YEL069C, YNR072W | HXT13, HXT17 | 1.46 | 2.34 | 0.36657784 |
| YHL035C | VMR1 | 1.45 | 0.87 | 0.14859112 |
| YKL161C | KDX1 | 1.42 | 0.39 | 0.04934333 |
| YPR078C | --- | 1.42 | 0.36 | 0.00868702 |
| YKL120W | OAC1 | 1.41 | 0.54 | 0.01183173 |
| YGR204C-A | --- | 1.41 | 1.24 | 0.01785192 |
| YGL224C | SDT1 | 1.41 | 0.84 | 0.00101731 |
| YBR148W | YSW1 | 1.40 | 0.46 | 0.08312594 |
| YLR205C | HMX1 | 1.40 | 0.58 | 0.07670162 |
| YBR301W | PAU24 | 1.40 | 0.05 | 0.02729238 |
| YGL125W | MET13 | 1.40 | 0.50 | 0.00150402 |
| YIL116W | HIS5 | 1.38 | 0.18 | 0.01325996 |
| YPL250C | ICY2 | 1.38 | 0.55 | 0.00059314 |
| YHR048W | YHK8 | 1.38 | 0.11 | 0.03031932 |
| YOL064C | MET22 | 1.36 | 0.36 | 0.01465976 |
| YPL264C | --- | 1.36 | 0.50 | 0.00238525 |
| YBL005W-A (+ Homologs) | --- | 1.35 | 0.25 | 0.04921427 |
| YBR284W | --- | 1.35 | 0.69 | 0.13582784 |
| YIR034C | LYS1 | 1.35 | 0.54 | 0.00506078 |
| YHL044W | --- | 1.33 | 0.80 | 0.00121048 |
| YFR030W | MET10 | 1.33 | 0.49 | 0.07926356 |
