# Supplementary material for: Polyglutamine toxicity in yeast induces metabolic alterations and mitochondrial defects
Source: BMC Genomics. 2015 Sep 3;16(1):662. doi: 10.1186/s12864-015-1831-7 (PMC4558792; doi:10.1186/s12864-015-1831-7)
Supplement: Additional file 10: — Genes with increased expression in Q 30 -YFP colonies versus Q 0 -YFP colonies. The table summarizes the expression differences of two data sets (Q0_3d, Q30_3d). Standard deviation and p-values were obtained as described above. (DOCX 16 kb) [file 12864_2015_1831_MOESM10_ESM.docx]

**Additional file 10: Genes with increased expression in Q_30_-YFP colonies versus Q_0_-YFP colonies.**

| **ID** | **log_2_ Q_30_/Q_0_** | **Standard deviation** | **p-value** |
| --- | --- | --- | --- |
| YDL186W  YJL043W  HXT15  AIM26  YML100W-A  YHR213W  FYV12  YJR005C-A  YBL100W-C  HXT1  YER175W-A  YGR273C  YOR268C  SPO13  YLR162W  YGL235W  YGR204C-A  YAR075W  YKL096C-B  YGL188C-A  YBL071C-B  IME4  YGL262W  YHL015W-A  YLR154W-F  YCL042W  HXT3  YOL164W-A  YEL075C  YOL166W-A  YGR174W-A  YGL006W-A  ANS1  IZH4  COS12  ALD3  HXT13  YKR075C  YMR175W-A  SOL1  DCV1  HXT17  YML054C-A  LYS9  REC114  YBR182C-A  YRF1-6  YDR246W-A  YNR066C  YLR031W  YNL234W  YBR184W  DAL4  STL1  PAI3  YIL029C  YAL016C-B  YKL107W  NFT1  MET17  HXT13  YNR071C  GCV1  COS6  YHR177W  SER3 | 2,50  2,44  2,19  2,18  2,07  1,95  1,93  1,87  1,80  1,68  1,65  1,63  1,42  1,38  1,29  1,24  1,20  1,16  1,16  1,14  1,12  1,09  1,08  1,07  1,06  1,06  1,06  1,05  1,04  1,85  0,29  0,98  0,93  0,89  0,87  0,86  0,65  0,79  0,78  0,77  0,76  0,76  0,76  0,76  0,76  0,75  0,73  0,73  0,72  0,72  0,71  0,70  0,70  0,69  0,69  0,69  0,69  0,67  0,66  0,65  0,65  0,64  0,64  0,62  0,62  0,61 | 2,18  4,67  3,49  0,83  3,75  3,17  1,88  3,15  4,03  2,26  1,57  2,58  2,25  0,69  0,75  2,66  1,62  2,91  1,10  0,68  1,93  2,18  1,35  3,91  2,16  1,27  1,31  0,71  0,12  2,65  0,63  1,53  2,32  1,60  1,53  0,60  1,54  0,53  0,69  0,69  1,23  1,09  1,32  0,80  1,22  0,55  0,56  2,76  0,97  0,53  0,96  0,83  1,46  0,57  0,67  0,91  0,33  1,01  1,10  1,05  1,54  0,98  0,52  0,38  0,86  0,78 | 0.153208681  0.416997651  0.344709804  0.061086859  0.400573872  0.364316143  0.169981751  0.374365508  0.184307551  0.490271632  0.327136827  0.207494187  0.364230604  0.365431857  0.118343547  0.160136  0.473836831  0.364756331  0.546409511  0.116590955  0.109010149  0.447628722  0.416665368  0.078335993  0.666744887  0.293430943  0.345342712  0.347649991  0.218966297  0.040281755  0.431611307  0.572988496  0.483917882  0.19334905  0.262132289  0.269341589  0.161388912  0.328988502  0.466139822  0.123122701  0.480902805  0.167004119  0.468182374  0.261796043  0.050027722  0.695653603  0.03687565  0.301178583  0.420965185  0.016991692  0.554754386  0.194977177  0.359690427  0.43278388  0.023739759  0.461384911  0.072388549  0.49490801  0.349335334  0.252577914  0.326849597  0.037080335  0.130371774  0.332718907  0.206686614  0.66688915 |
