# Supplementary material for: Polyglutamine toxicity in yeast induces metabolic alterations and mitochondrial defects
Source: BMC Genomics. 2015 Sep 3;16(1):662. doi: 10.1186/s12864-015-1831-7 (PMC4558792; doi:10.1186/s12864-015-1831-7)
Supplement: Additional file 13: — Peak assignments in the 13 C-NMR spectra. Peaks as observed in the 13C NMR spectra were assigned with the help of databases and literature. (DOCX 14 kb) [file 12864_2015_1831_MOESM13_ESM.docx]

**Additional file 13: Peak assignments of the ^13^C-NMR spectra.**

| **Position (ppm)** |  | **Peak label** | **Attributed to** |
| --- | --- | --- | --- |
| 16.7  57.4  27.6  169.9  205.3  60.5  69.5  74.2  75.8  92.1  95.9  62.5  72.0  160.3  124 | Duplett  Duplett  Duplett of Dupletts  Duplett of Dupletts  Duplett of Dupletts  Triplett  Triplett  Duplett  Duplett  Duplett  Triplett  Singlett | Et  Pyr  G  Glyc  Carbonate | Ethanol  Pyruvate  Glucose  Glycerol  Carbonate  unassigned |
