# Supplementary material for: Polyglutamine toxicity in yeast induces metabolic alterations and mitochondrial defects
Source: BMC Genomics. 2015 Sep 3;16(1):662. doi: 10.1186/s12864-015-1831-7 (PMC4558792; doi:10.1186/s12864-015-1831-7)
Supplement: Additional file 16: — GFP-strains used in this manuscript. For the analysis of cellular structures the respective strains of the Yeast GFP fusion collection in the background of EY0986 (MAT a; his3Δ1; leu2Δ0; met15Δ0; ura3Δ0) were deployed and transformed with either p Q0 or p Q56 [59]. Sul1 was not investigated further as it only shows very faint fluorescence. (DOCX 14 kb) [file 12864_2015_1831_MOESM16_ESM.docx]

**Additional file 16: Genomic GFP-marker strains.**

| **Name** | **Gene** |
| --- | --- |
| Cox4 | YGL187C |
| Met10 | YFR030W |
| Met13 | YGL125W |
| Met22 | YOL064C |
| Met28 | YIR017C |
| Om45 | YIL136W |
| Sul1 | YGL254W |
| Tmt1 | YER175C |
